# Human herpesvirus 8 – A novel human pathogen

**DOI:** 10.1186/1743-422X-2-78

**Published:** 2005-09-02

**Authors:** Daniel C Edelman

**Affiliations:** 1 University of Maryland Baltimore, School of Medicine, Department of Pathology, 725 West Lombard Street, Rm. S407, Baltimore, Maryland 21201, USA

## Abstract

In 1994, Chang and Moore reported on the latest of the gammaherpesviruses to infect humans, human herpesvirus 8 (HHV-8) [1]. This novel herpesvirus has and continues to present challenges to define its scope of involvement in human disease. In this review, aspects of HHV-8 infection are discussed, such as, the human immune response, viral pathogenesis and transmission, viral disease entities, and the virus's epidemiology with an emphasis on HHV-8 diagnostics.

## 1. The Herpesviruses

### 1.A. Classification of herpesviruses

More than 100 herpesviruses have been discovered, of which all are double-stranded DNA viruses that can establish latent infections in their respective vertebrate hosts; however, only eight regularly infect humans. The *Herpesvirinea *family is subdivided into three subfamilies: the *Alpha-*, *Beta-*, or *Gammaherpesvirinea*. This classification was created by the Herpesvirus Study Group of the International Committee on Taxonomy of Viruses using biological properties and it does not rely upon DNA sequence homology. However, researchers have been able to identify and appropriately characterize the viral subfamilies using DNA sequence analysis of the DNA polymerase gene; other investigators have been successful using the glycoprotein B gene [2].

The *Alphaherpesvirinea *are defined by variable cellular host range, shorter viral reproductive cycle, rapid growth in culture, high cytotoxic effects, and the ability to establish latency in sensory ganglia. In humans, these are termed herpes simplex viruses 1 and 2 (HSV-1 and HSV-2) and varicella zoster virus (VZV), and represent human herpesviruses 1, 2, and 3 [2].

The *Betaherpesvirinea *have a more restricted host range with a longer reproductive viral cycle and slower growth in culture. Infected cells show cytomegalia (enlargement of the infected cells). Latency is established in secretory glands, lymphoreticular cells, and in tissues such as the kidneys among others. In humans, these are termed human cytomegalovirus (HCMV or herpesvirus 5), human herpesviruses 6A and 6B (HHV-6A and -6B), and human herpesvirus 7 (HHV-7). HHV-7 has also been called the roseolavirus, after the disease roseola infantum it causes in children [2].

The *Gammaherpesvirinea *have a host range that is found within organisms that are part of the Family or Order of the natural host. In vitro replication of the viruses occurs in lymphoblastoid cells, but some lytic infections occur in epithelial and fibroblasts for some viral species in this subfamily. Gammaherpesviruses are specific for either B or T cells with latent virus found in lymphoid tissues. Only two human Gammaherpesviruses are known, human herpesvirus 4, referred to as Epstein-Barr virus (EBV), and human herpesvirus 8, referred to as HHV-8 or Kaposi's sarcoma-associated herpesvirus (KSHV) [2]. The gammaherpesviruses subfamily contains two genera (a classification of closely related viruses) that includes both the gamma-1 or *Lymphocryptovirus *(LCV) and the gamma-2 or *Rhadinovirus *(RDV) virus genera. EBV is the only LCV and HHV-8 is the only RDV discovered in humans. LCV is found only in primates but RDV can be found in both primates and subprimate mammals. RDV DNAs are more diverse across species and are found in a broader range of mammalian species. It is thought that RDVs evolved before LCVs [2].

HHV-8 has sequence homology and genetic structure that is close to another RDV, *Herpesvirus saimiri *(HVS) [3]. HVS can cause fulminant T-cell lymphoma in its primate host and can immortalize infected T-cells [4]. Rhadinaviruses can infect ungulates, mice, and rabbits and all share a particular genomic organization characterized by large flanking, highly repetitive DNA repeats of high G/C content [5].

### 1.B. The phenotypic structure of herpesviruses

The phenotypic architecture of the *Herpesviridae *family viruses characterizes these viruses. Customarily, herpesviruses have a central viral core that contains a linear double stranded DNA. This DNA is in the form of a torus, exemplified by a hole through the middle and the DNA is embedded in a proteinaceous spindle [6]. The capsid is icosadeltahedral (16 surfaces) with 2-fold symmetry and a diameter of 100–120 nm that is partially dependent upon the thickness of the tegument. The capsid has 162 capsomeres. The three dimensional structure of the HHV-8 capsid was determined by cryo-electron microscopy (EM) and was found to be composed of 12 pentons, 150 hexons, and 320 triplexes arranged as expected in the icosadeltahedral lattice with 20 faces; the capsids are 125 nm in diameter [7]. Transmission EM showed a bulls-eye appearance in the virions with electron dense cores and amorphous teguments surrounding the viral core [8]. Interestingly, these structural characteristics were seen in endemic KS lesions as early as 1984, but were not recognized at that time as the possible etiology of the disease [9].

The herpesvirus tegument, an amorphorous proteinaceous material that under EM lacks distinctive features, is found between the capsid and the envelope; it can be asymmetric in distribution. Thickness of the tegument is variable dependent upon its location in the cell and varies between different herpesviruses [10].

The herpesvirus envelope contains viral glycoprotein protrusions on the surface of the virus [2]. As shown by EM there is a trilaminar appearance [11] derived from the cellular membranes [12] and contains some lipid [13]. Glycoproteins protrude from the envelope and are more numerous and shorter than those found on other viruses. The presence of the envelope can influence the size measurement of the virus under EM conditions [2].

### 1.C. Genomic structure and genes of herpesviruses

There are six defined DNA genomic sequence arrangements for viruses in the *Herpesviridae *family. Of the human herpesviruses, EBV and HHV-8 are in class C. In this grouping, the number of direct terminal repeats are smaller than for other herpesviruses and there are other repeats found within the genome itself that subdivide the genome into unique stretches [2]. All known herpesviruses have capsid packaging signals at their termini [14].

The majority of herpes genes contain upstream promoter and regulatory sequences, an initiation site followed by a 5' nontranslated leader sequence, the open reading frame (Orf) itself, some 3' nontranslated sequences, and finally, a polyadenylation signal. There are exceptions to this format because initiation from an internal in-frame methionine has been reported [15].

Gene overlaps are common, whereby the promoter sequences of antisense strand (3') genes are located in the coding region of sense strand (5') genes; Orfs can be antisense to one another. Proteins can be embedded within larger coding sequences and yet have different functions. Most genes are not spliced and therefore are without introns and sequences for noncoding RNAs are present [2].

Herpesviruses code for genes that code for proteins involved in establishment of latency, production of DNA, and structural proteins for viral replication, nucleic acid packaging, viral entry, capsid envelopment, for the blocking or modifying host immune defenses, and transitions from latency to lytic growth. Although all herpesviruses establish latency, some (e.g., HSV) do not absolutely require latent protein expression to remain in latency, unlike others (e.g., EBV and HHV-8). Herpesviruses can alter their environment by affecting host cell protein synthesis, host cell DNA replication, immortalizing the host cell, and the host's immune responses (e.g., blocking apoptosis, cell surface MHC I expression, modulation of the interferon pathway) [2].

Gene expression is occurs in two major stages: latency and lytic growth. In the latent phase, there can be replication of circular episomal DNA, and latency typically involves the expression of only a few latently expressed genes. Generally, most host cells infected by herpesviruses exist in a latent phase. When KS tissue or BCBL-1 HHV-8 infected cultured cells are analyzed [8], the vast majority of the infected cells are infected with latent HHV-8 virus. Only a small percent of the cells (≤ 1%) appear to be undergoing lytic replication in a latently infected cell line [16].

The herpesvirus lytic replicative phase can itself be divided into four stages:

1. α or immediate early (IE), which requires no prior viral protein synthesis. In the IE stage, genes involved in trans-activating transcription from other viral genes are expressed.

2. β or early genes (E), whose expression is independent of viral DNA synthesis.

3. Following the E phase, γ1 or partial late genes are expressed in concert with the beginning of viral DNA synthesis.

4. γ2 or late genes, where viral protein expression is totally dependent upon synthesis of viral DNA and where the expression of virion structural genes encoding for capsid proteins and envelope glycoproteins occurs.

#### 1.C.a. Genomic structure and genes of HHV-8

In the viral capsid, HHV-8 DNA is linear and double stranded, but upon infection of the host cell and release from the viral capsid, it circularizes. Reports of the length of the HHV-8 genome have been complicated by its numerous, hard-to-sequence, terminal repeats. Renne et al. [17] reported a length of 170 kilobases (Kb) but Moore et al. [18] suggested a length of 270 Kb after analysis with clamped homogeneous electric field (CHEF) gel electrophoresis. Base pair composition on average across the HHV-8 genome is 59% G/C; however, this content can vary in specific areas across the genome [2]. HHV-8 possesses a long unique region (LUR) at approximately 145 Kb, with at least 87 genes, flanked by terminal repeats (TRs). Varying amounts of TR lengths have been observed in the different virus isolates. These repeats are 801 base pairs in length with 85% G/C content, and have putative packaging and cleavage signals [19]. The LUR is similar to HVS and the HHV-8 genes are named after their HVS counterparts. New genes are still being discovered through transcription experiments with alternative splicing; the initial annotation by Russo et al. [19] was purposely conservative. A "K" prefix denotes no genetic homology to any HVS genes (K1–K15).

HHV-8 possesses approximately 26 core genes, shared and highly conserved across the alpha-, beta-, and gammaherpesviruses. These genes are in seven basic gene blocks, but the order and orientation can differ between subfamilies. These genes include those for gene regulation, nucleotide metabolism, DNA replication, and virion maturation and structure (capsid, tegument, and envelope). HHV-8, being a gammaherpesvirus, encodes more cellular genes than other subfamily viruses. HHV-8 in particular, has a large arrangement of human host gene homologs (at least 12) not shared by other human herpesviruses [19]. These genes seemed to have been acquired from human cellular cDNA as evidenced by the lack of introns. Some retain host function or have been modified to be constitutively active; an example of this is the viral cyclin-D gene [20]. Cellular homologs related to known oncogenes have been identified in HHV-8, including genes encoding viral Bcl-2, cyclin D, interleukin-6, G-protein-coupled receptor, and ribonucleotide reductase [19]. Other genes, such as the chemokine receptor ORF 74, have homologues in other members of the RDV genera [19]. A number of other genes derived from the capsid of HHV-8 have been identified, including Orf 25, Orf 26, and Orf 65 [19]. In addition to virion structural proteins and genes involved in virus replication, HHV-8, typical of a herpesvirus, has genes and regulatory components that interact with the host immune system, presumably as an antidote against cellular host defenses [21].

HHV-8 gene expression has been classified into three stages by current investigators, unlike the four stages of other herpesviruses described above [22]. Class I genes are those that are expressed without the need for chemical induction of the viral lytic phase. Class II genes are induced to increased levels after chemical induction. However, Class III genes, are only expressed after chemical induction.

### 1.D. The biology of HHV-8

HHV-8 shares four main biological properties with other herpesviruses:

1. A broad array of enzymes involved in nucleic acid metabolism, DNA synthesis, and protein processing.

2. DNA synthesis and capsid formation occur in the nucleus of the host cell and the viral capsid is enveloped at the nuclear membrane.

3. Production of infectious progeny virus in the lytic phase can kill the host cell.

4. The virus can attain a latent state in the host cell with closed circular episomes and a minimal amount of gene expression. Latent genomes, however, can become lytic with the proper stimulation using chemical agents such as sodium butyrate [2].

Several human host cells are permissive for HHV-8 infection. Two prototype cells are the B-cells of the body-cavity-based lymphoma (BCBL) or pleural effusion lymphoma (PEL) [23] and the spindle cells characteristic of Kaposi's sarcoma (KS) [24]. Renne et al. [25] surveyed 38 mammalian cell lines or cell types and was only able to detect by RT-PCR the presence of infectivity from BCBL-1 derived virions in 11 of the 38. However, at least one cell type from lymphoid, endothelial, epithelial, fibroblastoid, and cancer cell types was permissive for infection. The 293 human kidney epithelial cell line was most susceptible in that study [25]. Natural cellular reservoirs for HHV-8 are CD19+ B-cells [26]. Natural infection in other cell types have been reported for endothelium [27], monocytes [28], prostate glandular epithelium [29], dorsal root sensory ganglion cells [30], and spindle cells of KS tumors [27].

Like other rhadinoviruses, HHV-8 might only be pathogenic when other cofactors are involved, such as concurrent infection with HIV or in an immunocompromised host. In the natural healthy host, the virus is relatively benign [5], however, currently, there is no known host other than humans.

### 1.E. Comparisons of HHV-8 to other herpesviruses

LCV (EBV) and RDV (HHV-8) genomes are more closely related to each other than to the alpha- and betaherpesviruses [18]. HHV-8 does not immortalize B-cells in vitro, as does EBV. HHV-8 has similar large reiterations of the TR as found with EBV but lack EBV's long internal repeats. HHV-8 possesses genes coding for dihydrofolate reductase (DHFR), interferon regulatory factor (IRF), G-protein coupled receptor (GPCR), chemokine analogs, and cyclin-D that are absent from the EBV genome [19]. Fifty-four of 75 HHV-8 genes are collinear with their EBV homologs. Among these 54 genes, the average amino acid identity is 35%. EBV has three forms of viral latency but HHV-8 has only one that has been identified.

### 1.F. Serodiagnostics of other herpesviruses

#### I.F.a. *Alphaherpesvirinea*

HSV infection is optimally detected through direct culture of tissues or secretions with observation of cytopathic effect (CPE) usually occurring in animal embryo cells after 1 – 3 days. Sensitivity of detection of infection is dependent upon the stage of the clinical illness with an average sensitivity of approximately 80%. The shell vial technique, a modified immunofluorescent assay, is also used. VZV grows with more difficulty in culture and it takes 4 to 8 days until CPE is evident, but shell vial techniques can improve the ability to detect VZV infection. Immunofluorescent assay detection (IFA) using monoclonal antibodies (mAb) and using samples taken from the lesions is much quicker than culture methods. However, serology has not been employed conventionally due to the successful culturing techniques. Also, for a successful serological diagnosis, serology requires acute and convalescent samples. Neither culture nor serology has shown optimal sensitivity. Detection of specific glycolsylated proteins can distinguish HSV-1 from HSV-2 infection [2].

#### I.F.b. *Betaherpesvirinea*

These viruses (HCMV, HHV-6 & 7) have a more restricted host range than the alpha herpesviruses and exhibit slower growth in culture. They are ubiquitous in the general population but cause serious disease in immunocompromised patients. Diagnosis is difficult due to the absence of clinical disease in healthy persons; virus can be present without pathological effect in humans [2].

Current diagnosis of HCMV is complicated by the intrinsic labiality of the virus and that CPE is not seen in human fibroblast culture cells until after one to three weeks of growth. However, shell vial assays can give results in 24 – 48 hours [2]. The presence of HCMV in peripheral blood is diagnostic for infection even if found in otherwise healthy patients without clinical symptoms. Detection of the HCMV protein, pp65, by an antigen assay is commercially available and can be used for rapid diagnosis of HCMV infection. The pp65 antigen comes from the HCMV lower matrix phosphoprotein customarily found in white blood cells. This antigen test has better sensitivity than culture and can provide positive laboratory results in a few hours. A mAb is used to detect pp65, but the antigen is labile and laboratory tests need to be run within 24 hours of the blood collection [2]. HCMV IgM antibody is diagnostic for HCMV infection in the context of mononucleosis-like disease where the patient is EBV negative. However, acute EBV infection can produce a false positive HCMV IgM test result [31].

For HHV-6 and 7, asymptomatic viral shedding is common in the benign carrier state. Culture of these viruses has been successful with umbilical cord lymphocytes, but there is high background. There are a lack of diagnostic criteria to interpret serologic test results in immunocompromised patients, although the finding of seroconversion in infants is diagnostic [2]. The IFA test using virally infected cells has been commonly used with success [32].

#### I.F.c. *Gammaherpesvirinea *and associated antigens

EBV replicates *in vivo *in lymphoid and epithelial cells and can be cultured in immortalized umbilical cord lymphocytes; EBV antigen is found within the cells. Serology is used for diagnosis of infectious mononucleosis (IM) by detecting IgM heterophile antibodies that agglutinate with red blood cells of horses. Serologic assays can also measure antibodies to the EBV viral capsid antigen (VCA) that is composed of four different proteins, the early antigens (EA) of which there are five proteins, and the nuclear antigens (NA). Testing for IgM against VCA defines acute infection and corresponds to clinical sequelae but lasts only a few months; however, IgG remains for the life of the patient [33]. Anti-EA antibodies arise within a few weeks but are not detectable in all patients with mononucleosis [33]. Anti-NA antibodies arise after the advent of EA antibodies and persist for life [33]. In contrast to acute infection, serology is not useful for post-transplant lymphoproliferative disorder (PTLD) and antigen detection or detection by PCR of viral nucleic acids is required [2]. Antibody production might be compromised due to the host's immunocompromised state or the rapid growth of the polyclonal tumor prior to reactivation of the memory immune response. Antigenic cross reactivity between EBV and other human herpesviruses is rare [2]. This is demonstrated in one study of 42 patients with nasopharyngeal carcinoma, known to be associated with EBV and of all persons positive for EBV VCA, only two showed reactivity to HHV-8 lytic proteins [34].

The humoral antibody response to EBV infection is against four serologically defined antigens [2]:

1. Epstein – Barr virus NA (EBNA) in latently infected cells.

2. EA either in its diffuse (methanol resistant) or restricted (methanol sensitive) compartments, expressed early in the viral lytic cycle.

3. VCA found during the late lytic cycle.

4. Membrane antigen (MA; gp350) as part of the viral envelope and is found on the surface of cells in the lytic phase. Anti-MA antibody levels correlate well with neutralization of the virus.

These EBV antigens are composites of several distinct proteins; e.g. EBNA = EBNA 1, 2, 3A, 3B, 3C. LP and EBNA1 are the most antigenic. The detection of EBV in IM is based upon the use of an enzyme-linked immunosorbant assay (ELISA) to detect IgM specific to BALF2 and BMRF1, the EA antigens, or against VCA components BFRF3 and BLRF2; combinations of these antigens are still recommended [35,36]. Diagnostics of HHV-8 will be discussed at length in Section 8, HHV-8 Diagnostics.

## 2. HHV-8 Immune Responses and Infectivity

As a prelude to the discussion about HHV-8 immune responses, antibody responses in primary EBV infection are presented as a contrasting system. Upon the appearance of clinical symptoms after EBV infection, most patients have rising IgM antibody titers to VCA and EA; IgA titers are transient [37]. The IgM anti-VCA response disappears over the next few months but the IgG titer falls to a steady state after previously peaking. In comparison, anti-EA IgG titers fall faster and can disappear entirely [2]. Many patients show an EBNA2 IgG response during the acute phase, but an EBNA1 IgG response usually does not appear until convalescence [38]. This delayed EBNA1 response is probably not due to the delay in immune recognition of the latently infected cells or of the released latent antigen because EBNA2 is recognized shortly after infection. Possibly EBNA1 is expressed at a later time point in the virus's life cycle. Latent membrane protein-1 (LMP-1) and LMP-2 antibody responses are rare [39].

Anti-gp350 or membrane antigen (MA) IgM antibodies are neutralizing with the IgG response arising only much later in the infection. These neutralizing antibody (nAb) titers tend to reach a plateau and stay at that level for long periods of time [37]. IgG, IgM and IgA levels are elevated universally in the human host upon EBV infection due to the general activation of B-cells [2]. In addition, heterophile antibodies and autoantibodies, mostly of the IgM class, show a transient increase in titer during acute infection.

In persistent EBV infection, healthy infected individuals are consistently anti-VCA IgG, anti-MA neutralizing antibody positive, and anti-EBNA1 positive. Titers can vary greatly among individuals, but these differences are consistently relative over time [2]. It is unknown why different antibody responses exist for EBV infection.

In general, after herpesvirus infection, some patients present with IgM levels that can be transient or at a low level for varying periods. These can last for up to a year making it difficult to gauge recent infection based upon IgM reactivity alone. In addition, IgM can be detected in viral reactivations [2]. An example of this is found with VZV, which shows an IgM response upon reactivation [40].

### 2.A. The neutralizing antibody immune response to HHV-8

Neutralizing antibodies are part of the humoral defense system against viral infection. The presence of nAb has been detected by searching for the effect of inhibition by nAb against HHV-8 viral infection in transformed dermal microvascular endothelial cells [41]. By quantifying the level of viral infection by indirect immunofluorescence assay (IFA), inhibition of infection was determined by comparing the level of infection in cells obtained with HHV-8 seropositive sera as compared to the level shown by incubation with seronegative sera. When the seropositive sera was diluted at 1:10 or 1:50 there was significant inhibition compared to the seronegative controls (*P *= 0.036). However, at a 1:500 dilution, the inhibitory effects of the sera disappeared. The nAb were found in the IgG fraction as shown by depletion of IgG antibody with protein A, which reversed the inhibitory effect.

Similarly, the presence and effect of nAb in the context of HHV-8 infection were investigated by measuring the infectivity in the 293 culture cell line [42]. Kimball et al. also discovered that the nAb were found in the IgG fraction and that compliment was not required for the neutralization. Importantly, their study found that those patients with KS had significantly lower nAb titers than other groups, independent of their HIV status. This suggested a possible role for nAb in the prevention of progression from latent asymptomatic HHV-8 infection to KS disease. They state that the positive effects of nAb were independent of CD4+ counts.

In contrast to these two reports, Inoue et al. observed the effects of nAb action, but concluded that nAb do not affect the progression to KS [43]. These antibodies were found in both KS+ and KS- groups with prevalences of 24% and 31%, respectively, but there was no significance in the difference (*P *= 0.64). This conflicting finding could perhaps be explained by the specific cohorts used. Other possibilities are the use by Inoue et al. of a colorimetric reporter system and their choice of cutoff at 30% neutralization; where as Kimball et al. used 50% inhibition as the cut off [42]. Additional discussion of HHV-8 antibody responses can be found in Sections 7 and 8.

### 2.B. Cytologic immune responses to HHV-8

Cell mediated immunology studies of HHV-8 have indicated that there are specific cytotoxic T-lymphocyte (CTL) responses against the virus. In an investigation of five cases of HIV negative subjects that seroconverted to HHV-8, Wang et al. explored the CD8+ T-cell response to five HHV-8 lytic proteins and found that CD8+ T-cells are involved in the control of primary HHV-8 infection [44]. They found that there were no major changes in the numbers of T-cell phenotypes or activation of T-cells, which differed from primary EBV infection that usually produces global increases in the numbers of T-cells. There was also no suppressive effect on other T-cell specificities as seen with EBV infection. They observed distinct CD8+, HLA class I restricted responses and increases in the interferon-gamma (IFN-γ) response to at least three of the five lytic antigens in each of the five subjects. No antigen was dominant in the elicited T-cell response. They observed that HHV-8 antibody titers to lytic IFA proteins paralleled the cytolytic responses. The CD8+ reactivity declined after several years possibly because of the lack of stimulation; the normal biology of HHV-8 is to enter a more latent state after primary infection. More T-cells produced a response of INF-γ production as opposed to CTL precursor production, but neither response was as strong as that observed when the T-cells were challenged with the HCMV pp65 antigenic protein. Osman et al. investigated HLA class I restricted CTL activity directed against the HHV-8 K8.1 lytic antigen [45]. They also investigated an additional lytic protein (K1) and one latent protein (K12) as antigens. Chromium release assays showed that CTL reactivity was detected against all three proteins, but not every patient had reactivity to all three antigens. Specific HLA alleles were able to present more than one of the viral proteins; e.g., HLA B8 could present all three antigens. Most patients with KS and were HIV+ did not have CTL responses indicative of compromised cellular immune systems. In one patient, whose KS had resolved under HAART therapy, CTL activity was restored. In general, these investigators showed that higher titers against HHV-8 LANA1 (Orf 73), i.e., more severe KS, correlated with less CTL response.

In a study of seroconversions in Amsterdam, Goudsmit et al. found that CD4+ T-cell levels did not affect the rate of seroconversions, but once HHV-8 infection had occurred, a decline in CD4+ cells was associated with increasing reactivity against the Orf 65 antigen [46]. Similar findings have been reported by Kimball et al. where persons with KS have higher levels of anti-HHV-8 antibodies and lower CD4+ counts than those without KS, but where both populations have HIV infection [42]. This suggests that viral replication had increased in the context of a more limited CD4 response. Recent investigation [47] has shown that NK cell function is important for the control of latent HHV-8 infection and abrogation of this important immune response can lead to more progressive KS disease.

### 2.C. Reactivation of HHV-8 infectivity

Using peripheral blood mononuclear cells (PBMCs) culled from KS patients and grown in culture, Monini et al. showed that reactivation of HHV-8 required at least the inflammatory cytokine (IC) INF-γ [48]. They observed that both B-cells and monocytes latently infected with HHV-8 responded to this IC with induction of lytic replication. They proposed that increases in HHV-8 viral load are due to the reactivation of the virus after exposure to INF-γ. They also proposed that a likely scenario of KS pathogenesis is the recruitment of circulating monocytes into peripheral skin tissues, where upon exposure to ICs, their latent HHV-8 genomes enter into the lytic phase. The monocytes then rupture and free virus is available to infect local tissues. The monocytes might also differentiate into macrophages or spindle cells after exposure to the ICs and form the basis of latent HHV-8 infection in the tissues.

Reactivation is possible in the context of autologous peripheral blood stem cell transplantation. Luppi et al. [49] presented a case report that showed HHV-8 viral load in the serum of the transplant patient concomitant with fever, rash, diarrhea, and hepatitis some 17 days after the transplant. The patient had lytic antibodies before and after the transplant indicating a reactivation event.

### 2.D. Corporeal sites of HHV-8 infection

A number of studies [49-56] have investigated by molecular methods the presence of HHV-8 virions, as evidenced by the presence of viral DNA in body fluids and tissues of several at-risk populations (Table 1). PBMCs were the most commonly studied sample site, but a number of others, including serum or plasma, semen, saliva, and stool have been investigated (Table 1). PCR sensitivities were below 100 copies, although some studies used nested PCR [52] or Southern blotting [50].

**Table 1:** Compilation of select studies investigating the molecular presence of HHV-8 in different tissues and body fluids. KS, HIV+, and HIV- represent three populations at high, medium, and lower risk of HHV-8 infection, respectively.

	**KS Lesion**	**Normal Skin**	**PBMC**	**Plasma or Sera**	**Semen**	**Saliva**	**Feces**	**Other**
**KS+**	63/70 (90%)	17/57 (30%)	94/188 (50%)	33/151 (22%)	7/60 (12%)	26/71 (37%)	0/29	
**HIV+**		0/10	22/268 (8.2%)	5/164 (3.0%)	4/57 (7%)	9/87 (10%)		10/228 (4.4)
**HIV-**		0/1	3/381 (0.8%)	0/218	3/168 (1.8%)	7/108 (6.5%)		10/332(3.0)

At least four investigators used the K330 PCR as originally developed by Chang et al. [1]. Five articles described testing KS patients [50-52,54,55] and another five [50-52,55,56] compared HIV+ and HIV- subjects for the presence of HHV-8. Grandadam et al. [53] investigated multicentric Castleman's disease (MCD) in HIV+ patients and Luppi et al. [49] followed the unique case of a viral reactivation. For persons with KS, significant differences were found between sample sites; the HHV-8 prevalence was higher in KS lesions over that found in peripheral blood mononuclear cells (PBMCs), which were about equal in prevalence to saliva (Table 1). These three sites were better for finding the presence of HHV-8 rather than using plasma (*P *<10^-6^; *P *= 0.054; *P *≤ 0.02, respectively). For HIV+ persons, saliva and PBMCs were equivalent (*P *= 0.539) but both had a significant greater frequency of positive samples than were found in plasma (*P *= 0.016 and *P *= 0.031, respectively). Analysis of HIV- persons showed that saliva contained significantly more viral sequences than either PBMCs or plasma (*P *= 0.001 and *P *= 0.0006, respectively), which were commensurate with each other (*P *= 0.476).

It is noteworthy to add that several authors have observed the detectable presence of HHV-8 DNA to be intermittent [49,51,57,58]. Perhaps this has contributed to the overall lack of sensitivity of PCR in detecting HHV-8 infection. In keeping with this observation, Simpson et al. [59] stated, "...KSHV genomes were detected in peripheral blood monocyte DNA from KS patients less frequently than antibodies to either KSHV antigen in serum". Smith et al. [60] added that, "Overall, our serologic assay appeared more sensitive than PCR analysis of PBMC for the detection of HHV-8 infection". This last statement was reiterated by other authors (e.g. Angeloni et al. [61], Campbell et al. [62]). HHV-8 viremia is described at more length in Section 8, HHV-8 Diagnostics.

## 3. Pathogenic Mechanisms of HHV-8

The diversity of the HHV-8 genes allows the virus to assault and modulate its human host with many strategies. These pathogenic effects can promote active changes in the infected human host, such as to increase cytokine production or to suppress MHC Class I (MHC I) presentation of viral proteins to the immune system. The pathogenic activities that are due to HHV-8's unique K-series genes are summarized.

Interleukin-6 (IL-6) is a B-cell growth factor and its altered expression has been linked to several human diseases and malignancies, including MCD with its characteristic plasmacytosis and hypergammaglobulinemia. HHV-8 viral cytokine vIL-6 is encoded by the unique K2 gene, which exhibits 25% amino acid identity with the human homologue [63]. This viral gene is unique to HHV-8 among the other gammaherpesviruses and is the only HHV-8 encoded cytokine. It is a Class II transcript in that it is constitutively expressed in the BCP-1 cell line, but its expression is greatly increased after induction with TPA; it is a Class III transcript in the BC-1 cell line [63]. This feature of the protein implies that its pathogenic effects can be in the context of active viral infection. vIL-6 had activity on human myeloma cells [64], where exogenous application induced DNA synthesis and proliferation in the INA-6 myeloma cell line; this cell line is strictly dependent upon exogenous IL-6 for growth. Expression of vIL-6 mRNA transcripts was detected by in situ hybridization in tissue samples of KS, PEL, and MCD disease patients [65], demonstrating the in vivo expression of this cytokine. Staskus et al. showed that vIL-6 might be important in the pathogenesis of these three HHV-8 associated disorders, but the viral cytokine is variably expressed in the HHV-8 infected cells of these diseases [65]. For example, the number of vIL-6 copies in KS, PEL, and MCD cells was 10–100, 100–1000, and >1000 copies, respectively, per cell. Low levels of vIL-6 have also been observed in KS lesions by immunohistochemistry [63,66].

Several HHV-8 K-genes are active in modulating the adaptive immune response to HHV-8 infection. The K3 and K5 genes allow HHV-8 to evade detection by removing MHC I from the cell surface [21]. The proteins encoded by K3 and K5, MIR-1 and MIR-2, respectively, use a unique mechanism of enhanced endocytosis of the MHC I molecules and their subsequent degradation in lysosomes. MIR-2 protein also down regulates ICAM-1 and B7.2, accessory proteins necessary for proper T-cell stimulation [67].

The lack of MHC I on the cell surface can signal increased natural killer (NK) cell activity, but NK cells are modulated by the K13 gene product, v-FLICE inhibitory protein (vFLIP) [68]. Despite the Fas-dependent signaling (apoptosis triggering) caused by the NK cells, apoptosis is impaired because vFLIP binds to cellular procaspase-8 preventing its proteolytic cleavage into apoptotically active forms.

Another tactic to alter the cell-mediated response to HHV-8 infection is to make sure this response does not occur upon infection. HHV-8 creates a microenvironment where by there is preferential recruitment of T cell type 2 (Th2) lymphocytes with the release of IL-4 and IL-5 cytokines, which polarizes the immune response towards an antibody predominant immune reaction [69]. It is the Th1 response with the characteristic release of Inf-γ that stimulates cell-mediated immunity. Three HHV-8 chemokines, vCCL1, vCCL2, and vCCL3, also referred to as vMIP-1, vMIP-II, and vMIP-III, respectively, are encoded by the K6, K4, and K4.1 Orfs, respectively [70]. These chemokines activate Th2 responses through the CCR8, CCR3, and CCR4 receptors [70], respectively, but are antagonistic for the receptors that result in chemotaxis of Th1 and NK lymphocytes [71]. The vCCL3 is found in KS tumors and is thought to contribute to its pathogenesis [72]. Another HHV-8 gene, K14, encodes a neural cell adhesion-like protein (OX-2) that also promotes Th2 polarization and the production of inflammatory cytokines, such as IL-6 [73]. Other unique K-genes modify the immune system by interacting with the μ-chains of B-cell receptors and blocking transport to the cell surface (K1 or KIS) or by inhibiting interferon signaling (K9 or vIRF-1) [70]. The diverse repertoire of immune suppressive strategies exhibited by HHV-8 could explain the virus's success in establishing a high prevalence in populations where it is being actively transmitted, such as sub-Saharan Africa. However, it then brings into question why HHV-8 is not more successful in establishing infection in developed counties, even with people whose immune systems are compromised or constantly stimulated.

## 4. Transmission of HHV-8

Patterns of transmission for HHV-8 are being better defined as our understanding of the pathogenesis of this virus increases and testing methods are used strategically. The virus, first thought to be transmitted only sexually, is now also considered transmissible through low risk or more casual behaviors.

### 4.A. Sexual Transmission

The transmission of HHV-8 through sexual activities has been documented [74]; men with homosexual behaviors showed a 38% prevalence of HHV-8 as compared to 0% of men with no such activity. The increased prevalence correlated with the presence of sexually transmitted diseases (STD) and the number of male sexual partners. The presence of both HIV and HHV-8 produced a 10-year probability of 50% for developing KS [74].

Transmission from male genital secretions, specifically semen, is unlikely due to the low prevalence of detectable HHV-8 in semen samples obtained from both HIV+ or HIV- persons [52,55,56]. In a study of women with KS from Zimbabwe, between 28% and 37% had detectable HHV-8 DNA in their vaginal or cervical samples [75], but HHV-8 DNA was not found in any of the women without KS, even those with HHV-8 seropositivity. A possible explanation why perinatal transmission is infrequent in prevalence studies might be that transmission is limited to immunocompromised mothers where titers might be higher [75].

HHV-8 DNA is found most frequently and with increased viral burdens in saliva or other oral samples [56]. Sexual practices that include oral sex could therefore increase the possibility of transmission. Persons having STDs, such as syphilis and HIV, have an increased risk for greater HHV-8 prevalence [76]. However, in a study of 1,295 women in four USA cities, Cannon et al. did not find an association between the number of sex partners or engagement in commercial sexual practices to be a risk for increased HHV-8 prevalence [76].

### 4.B. Blood-borne transmission

Identification of HHV-8 in blood donors [58,77] has raised concern about the safety of the blood supply. Other reports [78] have tempered the concern of blood borne transmission after observing no transmission in 18 recipients of HHV-8 seropositive blood components. However, because of the small sample size, additional studies are required for this low prevalence population. In a multicenter study of 1,000 blood donors, approximately 3% of blood donors were considered seropositive, but none of the 138 total seropositive samples had detectable HHV-8 DNA in their PBMCs [79]. Without detectable virus, the possibility of infectious transmission seems remote.

However, blood-borne transmission seems to occur, but rarely. Two epidemiological markers for blood borne viral infection, HCV positivity and daily-injected drug use, were associated with increased HHV-8 infection in four large groups of women in the USA [76]. However, the overall prevalence of HBV and HCV among irregular drug users was higher than found with HHV-8, indicating a lower relative frequency of transmission of this herpesvirus.

Evidence that HHV-8 can be transmitted in populations of intravenous drug users (IVDU) and those HCV+, shows that transmission via blood is possible, albeit with difficulty [80]. Larger studies are required to determine if HHV-8 is a true threat to the blood supply. Such studies will be difficult to conduct due to the difficulty in detecting infectious virus in healthy individuals, the lack of culture methods to tests for cytopathic effect, and the anonymous nature of blood donations, which does not allow for follow up testing.

Important risk factors for transmission of the virus are a spouse's seropositivity and maternal seropositivity [81]. Although spousal seropositivity could include sexual transmission, transmission to children precludes this route, indicating more casual transmission is possible. Horizontal asexual transmission within families has been observed by other investigators [82]. Vertical transmission from mother to child at or before birth is also infrequent with few children from HHV-8 infected mothers showing HHV-8 sequences in their PBMCs at birth [83,84]. In a study of the presence of HHV-8 DNA in matched pairs of breast milk and saliva from the same mother, no HHV-8 sequences were found in the breast milk, but 29% of the saliva samples had HHV-8 DNA; therefore nursing of infants appears unlikely to be a route of infection [85], although, another study seemed to contradict this finding [86].

Of all anatomic sites, HHV-8 DNA is found most frequently in saliva, which also has higher viral concentrations than other secretions [56]. For this reason, it has been hypothesized that saliva could be the route of casual transfer of infectious virus among family members. It has been hypothesized that customarily licking an insect bite, such as from a mosquito, could transfer the virus [87].

### 4.C. Transplants

#### 4.C.a. Organ

Transmission of other herpesviruses (e.g., HCMV and HHV-6) has been documented [88] and the body of evidence is growing that HHV-8 disease after organ transplantation is a concern for the transplant physician. Most reports in the literature have presented data describing the prevalence and the possible ramifications of HHV-8 infection on donor kidney recipients.

However, the concern of HHV-8 transmission in the context of organ transplantation has two problems. First, there are no large studies of the donor's and the recipient's HHV-8 serostatus and presence of HHV-8 in donor blood and organ. Properly done, both antibody prevalence and a determination of infectious virus by PCR would be necessary. Follow up measuring possible seroreactivity every few months after transplant would be critical. Second, even once the problem is defined, there are no current establish procedures or parameters to monitor the patients both diagnostically and clinically; seemingly, both problems would have to be addressed in tandem.

In areas where endemic KS is not found and in normally healthy people, HHV-8 infection has not been shown to be a life threatening infection. However, in the context of immunosuppression, as with organ transplants, both primary infection and reactivation become a proven concern. Post-transplant immunosuppression can cause iatrogenic KS to appear [89]. The clinical significance of post-transplant KS can be rejection of the graft and death of the patient. In a study of 356 post-transplant patients with KS, 40% had visceral involvement, a manifestation of KS with poor prognosis, and 17% of those with visceral KS died from the tumor [89]. The KS tumor can recede after withdrawal of immunosuppressive therapy, but with immunological recovery, graft loss or organ impairment often emerges as a unwanted condition [89]. In an early study, Parravicini et al. [90] suggest that post-transplant KS is caused by emergence of latent HHV-8 after previously infected but clinically well transplant patients are immunosuppressed. Immunosuppression, such that occurs in transplant recipients, is known to facilitate reactivation of herpesviruses, (e.g., disseminated herpes zoster) and is associated with an increased incidence of herpesvirus associated lymphoproliferative malignancies [91].

Of importance, seroprevalence to HHV-8 increased from 6.4% to 17.7% overall one year after renal transplantation. In addition, seroconversion to HHV-8 occurred within the first year after renal transplantation in 25 of 220 patients and KS developed in two of the 25 within 26 months after transplantation [92]. KS developed within 20 months in two renal transplant recipients from the same cadaveric donor; Orf 73 genotyping confirmed that the virus was transmitted from the donor [93]. Detection of HHV-8 in the allograft kidneys or increases in antibody titer can be prognostic indicators of increased risk for KS [94]. Other studies have found the median time to KS from transplantation to be between 7 months [90] and 24 months [95].

In another study, the increased risk of acquiring HHV-8 infection was shown by 10% of 100 transplant patients who seroconverted to HHV-8, however, there was no pattern associated with the type of organ donated, and none of the donors that could be tested were seropositive [96]. Therefore the investigators concluded that the infection came from sources other than the transplanted organ; however this conclusion is lacking because healthy infected individuals (i.e., healthy organ donors) in the USA are less likely to exhibit antibodies, similar to blood donors, however, the organ might still harbor infectious virus or KS precursor cells [93,94].

In a comparison of kidney and liver transplants, seroconversion was observed in 12% of transplant patients, combined. The incidence of KS in kidney patients was higher than in liver recipients [97]. Importantly, patients already infected with HHV-8 had a greater chance to develop KS from viral reactivation than from primary infections [97]. In a large study of solid organ transplant recipients in Spain (n = 1,328), Munoz et al. [95] reported that the overall KS incidence was 1 in 200 with more males diagnosed with KS than females (6:1 ratio). High HHV-8 antibody titers or seroconversions were prognostic indicators of possible KS development.

Because increased prevalence in transplant patients might be due to reactivation of HHV-8 and the subsequent increase of antibody tiers [98], molecular methods, although normally less sensitive, would be better indicators of transmission. Another possibility would be the use of antibody avidity assays to detect highly avid antibodies that would be indicative of reactivation events [99].

Post-transplant KS can develop in the recipient from transmission of the virus from the donor to the recipient [93,94], and from KS progenitor cells seeded along with the donor organ, which undergo neoplastic change, and progress into KS [100]. HHV-8 DNA can be detected in the KS lesions from patients suffering from post-transplant cutaneous and visceral KS. Other organs without evidence of KS involvement can test positive for HHV-8 sequences [101], as can circulating spindle cells infected with HHV-8 [102]. Disease entities associated with HHV-8 in the context of transplantation continue to be discovered. In at least one report, investigators have suggested that EBV-negative post-transplant lymphoproliferative disorders (PTLD) might be caused by HHV-8 [103].

#### 4.C.b. Bone marrow/Peripheral blood stem cell

Non-neoplastic disease associated with HHV-8 has been documented [49,104]. Bone marrow failure was observed after a kidney transplant and after an autologous peripheral blood stem cell (PBSC) transplant for non-Hodgkin's lymphoma (NHL). HHV-8 produced a syndrome of fever, marrow aplasia and plasmacytosis; these occurred after primary infection and reactivation, respectively [104]. Neither patient presented with KS, but both had detectable HHV-8 sequences by PCR after transplantation and at the presentation of symptoms – both patients died. Another case report [49] showed reactivation of HHV-8 in a seropositive patient and documented nonmalignant disease 17 days after PBSC transplantation in the context of NHL. The patient presented with fever, cutaneous rash, diarrhea, and hepatitis; here too HHV-8 DNA was detected in the serum by PCR with higher viral loads with exacerbation of symptoms. Therefore, transplant patients who are HHV-8 positive could benefit from close clinical follow-up to preempt the occurrence of KS with judicious use of immune suppressive therapy or antiviral drugs, or to begin the early and therefore more effective treatment of the tumor once detected.

## 5. Diseases of HHV-8

HHV-8 poses challenging questions of diagnosis and pathology related to its role in the etiology of several human malignancies including KS, MCD, PEL, and possibly multiple myeloma (MM) and sarcoidosis, among others.

### 5.A. Primary infection

Identification of HHV-8 primary infection has been difficult due to the low incidence of infection in most populations studied, and because of the lack of known defining features. By using a diagnosis of exclusion and the temporal occurrence of symptoms and diagnostic criteria, limited studies have suggested several defining clinical sequelae of HHV-8 primary infection. In 15-year longitudinal study of >100 HIV negative men to study the natural history of primary HHV-8 infection, five cases of HHV-8 seroconversion were identified [44]. The effects of HHV-8 primary infection were explored in the absence of HIV coinfection and no debilitating disease was observed in the five seroconverters. Four patients exhibited clinical symptoms, which ranged from mild lymphadenopathy and diarrhea to fatigue and localized rash. These symptoms were significantly associated with HHV-8 seroconversion when compared to the 102 seronegative subjects who remained well.

Organ transplantation is another clinical setting for primary infection. In a patient receiving a renal transplant, bone marrow failure was associated with a syndrome of fever, marrow aplasia, and plasmacytosis [104]. The patient did not present with KS, but HHV-8 sequences were detected by PCR after transplantation and at the presentation of symptoms; the patient did not survive. This limited experience suggests that in the context of immunosuppression, primary infection can be lethal, but in healthy individuals, the infection presents with flu-like symptoms.

### 5.B. Kaposi's sarcoma

KS was first described by Moritz Kaposi in the 1870s [105] and was described as an aggressive tumor affecting patients younger than those currently observed. For all epidemiological forms of KS, the tumor presents as highly vascularized neoplasm that can be polyclonal, oligoclonal, or monoclonal. It's antigenic profile suggests either endothelial, lympho-endothelial, or macrophage origins [106]. Although the four epidemiological forms of KS have different clinical parameters, such as anatomic involvement and aggressiveness of the clinical course, they have HHV-8 infection in common with indistinguishable histopathology [107]. It is therefore believed that this transforming virus is the causative agent of KS and that HHV-8 fulfills Hill's criteria for causing KS [108,109].

HIV infection substantially increases the risk for development of KS, and therefore, the incidence of KS has increased substantially during the HIV pandemic, particularly in younger HIV-infected patients [110]. Striking differences in risk for acquiring AIDS-KS exist between different HIV transmission groups, varying from a high of 21% for homosexual men to a low of 1% for men with hemophilia. Women who acquired HIV infection by heterosexual contact with bisexual men were also at an increased risk for developing AIDS-KS [110]. Although the incidence of KS has decreased recently with the advent of highly active anti-retroviral (HAART) therapy, the appearance of drug resistant strains of HIV raises concern for a re-emergence of KS cases.

Browning et al., using a cell culture detection method, observed that the characteristic spindle cells of KS are present in the peripheral blood of patients presenting with KS; more importantly, these cells were found in the blood of HIV+ homosexual men, who are at higher risk for developing KS, than HIV+ IVDUs [102].

The first strong evidence that human herpes virus 8 (HHV-8) was the etiological agent of KS came from the use of a novel molecular technique, representational difference analysis (RDA) [1]. This complex molecular method identified viral molecular sequences in KS tumor tissue that were not present in paired normal tissue from the same individual [1]. The presence of nucleic acid sequences of the virus in tissues from all forms of KS [111] throughout the world, and the demonstration of antibodies to HHV-8 in KS patients from a number of serologic studies [112] has supported the association of this virus with KS. Because of its prominent association with KS, the virus is often referred to as Kaposi's sarcoma-associated herpesvirus or KSHV.

Proof of HHV-8's etiology in KS comes from the detection of HHV-8 nucleic acids in KS tissues but not in healthy tissues, from sero-epidemiological and molecular studies showing correlations to the risk of developing KS and progression of KS disease. The detection of antibodies to lytic HHV-8 antigens can be used as a predictor of development of KS [113]. Prospective studies of persons who subsequently developed KS, documented the appearance of infection more than 24 months prior to tumor development [114]. Data have shown that infection of primary endothelial cells with HHV-8 causes long term proliferation and transformation [115]. HHV-8 is detectable in the spindle cells of all forms of KS and in the nearby in situ endothelial cells [27].

#### 5.B.a. Classic KS

The classical or sporadic form of KS (CKS) is an indolent tumor affecting the elderly, preferentially men, in Mediterranean countries such as Italy, Israel, and Turkey [116]. The lesions tend to be found in the lower extremities and the disease, due to its non-aggressive course, usually does not kill those afflicted. HIV infection, unlike HHV-8, is not typically associated with CKS [117].

The older the age of the patient, the greater the risk of CKS disease progression; dissemination of KS lesions is more likely if immunosuppression also exists [118]. Certain behaviors, such as corticosteroid use and infrequent bathing were found to be risk factors for greater incidence of CKS but surprisingly, increased cigarette smoking actually lowered the risk [119]. The increased prevalence in Sardina of HHV-8 and CKS among family members of KS patients indicates that transmission of HHV-8 is probably by asexual routes [61].

#### 5.B.b. AIDS-KS

In the context of the acquired immunodeficiency syndrome (AIDS), KS is the most common malignancy and is an AIDS defining illness [120]. AIDS-KS is a more aggressive tumor than CKS and can disseminate into the viscera with a greater likelihood of death [121]. Unlike CKS, it presents more often multifocally and more frequently on the upper body and head regions [117].

In those with HIV infection, HHV-8 prevalence increases with higher risk of KS, and in patients with HHV-8 seroconversion there is a greater likelihood of KS development [74]. KS was more likely to develop when HHV-8 seroconversion occurred after the patient already had HIV [122,123]. An increased slope of CD4+ cell decline and higher HIV viral loads also suggested increased chances of KS development [122].

However, HIV infection alone might not be enough to increase the risk of KS. In a study of Ethiopians who had immigrated to Israel, only 0.85% of them with AIDS developed KS, as compared with 12.5% of non-Ethiopian AIDS patients (P < 0.001). The low risk of KS exists in the face of high HHV-8 prevalence (above 39%) in HIV+ and HIV- Ethiopian populations [124]. Clearly, other factors are necessary for KS development and ethnic or genetic protective factors might be involved.

#### 5.B.c. Endemic KS

HHV-8 was prevalent in Africa prior to the HIV epidemic, and therefore, was responsible for the large prevalence of KS seen on the continent before HIV changed the scope of KS presentations [125]. Prior to HIV coinfections, endemic KS affected men with an average age of 35 and very young children [126]. In Africa, endemic KS is found more often in women and children than in other areas of the world [125]. It presents in four clinical forms with one form similar to CKS, but found in younger adults; the other three forms are more aggressive, similar to AIDS-KS [117]. They vary in the age of presentation and the sites of involvement.

HIV coinfection has raised the prevalence of KS significantly in Africa. In Uganda, for example, prior to 1970, KS was diagnosed in no more than 7% of the male cancer population and in none of the female cancer population. However, by 1991, KS prevalence had risen to 49% in male cancer patients and to 18% in females [126]. The KS prevalence has increased in Africa, even in HIV negative populations, for unknown reasons [125].

Despite different clinical KS presentations, all forms of KS are associated with HHV-8 infection [111,127]. Paralleling the endemic KS pattern in children, HHV-8 infection in children is also high with seroprevalence reaching adult levels by the age of 20 and in certain locations even earlier [128]. This occurrence of horizontal infection in the young is similar to that seen with EBV in other continents [128]. Despite equal prevalences of HHV-8 in HIV-1 and HIV-2 patients, KS is found almost exclusively in persons infected with HIV-1 [129].

#### 5.B.d. Iatrogenic KS

More extensive information on transplant-associated KS and the involvement of HHV-8 can be found in the Literature Review: Section 4, Transmission of HHV-8. Briefly, iatrogenic KS can present either as a chronic condition or with a more rapid course [117]. Immunosuppression, such that occurs in transplant recipients, is known to facilitate reactivation of herpesviruses [91] and so too with HHV-8, transplant patients under immunosuppressive therapy can present with KS. Withdrawal of the therapy can cause the KS to regress [117].

Iatrogenic KS seems to vary in its geographic prevalences, perhaps reflecting the varying HHV-8 prevalence in the general populations of different countries [125]. KS appears most frequently in renal transplant patients [116] and in conjunction with cyclosporine treatment, used frequently in kidney transplant patients as an immunosuppressive drug; this steroid has been shown to reactivate HHV-8 in vitro [130].

### 5.C. Primary effusion lymphoma

First identified as a subset of body-cavity-based lymphomas (BCBL), PELs contain HHV-8 DNA sequences [23]. These lymphomas are distinct from malignancies that cause other body cavity effusions. PELs are characterized by several pathological features: 1) They do not exhibit Burkitt lymphoma-like morphology and do not have c-myc gene rearrangements; 2) They have a distinctive morphology comparable to large-cell immunoblastic lymphoma and anaplastic large-cell lymphoma; 3) They occur frequently in men; 4) They present initially as a lymphomatous effusion and remain localized to the body cavity of origin; 5) They express CD45 with frequent absence of B-cell associated antigens; 6) They exhibit clonal immunoglobulin gene rearrangements; 7) They can contain Epstein-Barr virus; 8) They lack oncogene rearrangements in genes such as bcl-2 and p53. Finally, patients with PELs, especially in the context of AIDS, invariably are infected with HHV-8 [23,131]. PEL cell lines have 50–150 copies of HHV-8 episomes per cell [8,132-136].

Divining the association of PELs with HHV-8's etiology has been difficult, because most PELs occur in the context of HIV infection, and the PELs account for only 0.13% of all AIDS malignancies in AIDS patients in the USA [137]. Importantly, PELs occur with an increased frequency in patients with prior KS [125]. In non-AIDS patients, the disease has been termed "classic" PEL by Ascoli et al. [138] where it presents in HIV negative patients, but with similar risk factors as CKS.

### 5.D. Multicentric Castleman's disease

HHV-8 has been found variably in association with MCD. MCD is a rare polyclonal B-cell angiolymphoproliferative disorder for which vascular proliferation has been found in germinal centers. It presents in heterogeneous forms both clinically and morphologically [139]. However, most of the B-cells in the tumor are not infected with HHV-8, and the HHV-8 infected cells are primarily located in the mantel zone of the follicle [140]. It is thought that uninfected cells are recruited into the tumor through HHV-8 paracrine mechanisms, such as vIL-6 [66], a known growth factor for the tumor. More than 90% of AIDS patients with MCD are HHV-8 positive, whereas MCD in the context of no HIV infection has a HHV-8 prevalence of approximately 40% [141]. Because of it rarity, MCD is difficult to closely associate statistically with HHV-8.

### 5.E. Other diseases

#### 5.E.a. Sarcoidosis

Sarcoidosis is a multisystemic granulomatous disease of unknown etiology that can involve many different organs such as the lungs, lymph nodes, and skin. Currently, a diagnosis can be established when clinical and radiological findings are confirmed by histological tests showing noncaseous granulomas in more than one tissue [142].

Di Alberti et al. reported that HHV-8 DNA was significantly more prevalent in pulmonary tissues, lymph nodes, skin and oral tissues in 17 Italian patients with sarcoidosis than in tissues from 96 control specimens [143]. However, a study by Belec et al. did not detect HHV-8 sequences in sarcoid tissues from French patients with systemic sarcoidosis [144]. Very little diagnostic HHV-8 serology has been reported on sarcoid patients. In one report, 18% of patients were seropositive, but the investigators concluded that this was not different from the observed prevalences in the patients' respective geographic regions [145].

#### 5.E.b. Multiple myeloma

There is debate concerning the etiology of MM. MM is the most common lymphoid cancer found in Blacks and the second most common in Caucasians [146]. It is a B cell malignancy of clonal origin in which the cancer cells, considered to be plasma cells, secrete monotypic immunoglobin. The pathogenesis of MM has been thought to include an initial antigenic stimulus of B cells followed by further mutagenic events. Studies have shown that autocrine and paracrine loops involving cytokines such as IL-6 [147], TNF, and IL-1β [148] are important as stimuli for growth of the MM cells. It has been believed that T cells and the bone marrow stroma are the sources of these cytokines. Three oncogenes have been implicated in MM; *ras*, *c-myc*, and *p53 *with prevalences of 30%, 25%, and 15–45%, respectively [146].

The possible role of HHV-8 in MM has been debated and a full report of the evidence is beyond the scope of this review. In brief, Rettig et al. [149] who originally reported that there was an association between the virus and the disease, investigated 15 MM patients along with eight patients presenting with monoclonal gammopathy of unknown significance (MGUS). They used PCR to amplify the KS330_233 _sequence of HHV-8 from bone marrow (BM) mononuclear and stromal cells of the MM patients. Southern blotting of the PCR fragments using an internal fragment confirmed the PCR results. They were able to amplify HHV-8 sequences from cultured BM stromal cells from 15/15 MM patients. However, none of the 23 non-cultured BM mononuclear preparations amplified. Said et al. [150] supported Rettig et al.'s claim that MM and HHV-8 were closely associated by finding 17 out of 20 BM biopsies from MM patients exhibiting HHV-8 positive cells. Gao et al. [151], provided important supportive serological evidence; of 27 MM patients, 81% and 52% possessed lytic and latent antibodies, respectively. All eleven patients with progressive MM were HHV-8 positive. The increased presence of lytic antibodies as opposed to latent antibodies was indicative of past or currently active viral infection in the MM patients.

Contrary to these findings, other groups have found a lack of supporting evidence. Whitby et al. [152] found latent antibodies in only 4/37 MM and in only 2/36 MGUS patients, but these prevalences were not significantly different from patients with Hodgkin's lymphoma, NHL, or normal blood donors. Additionally, whereas Rettig et al. postulated that MGUS might be the precursor of MM through infection with HHV-8 [149], Whitby and colleagues found that 4 persons with MGUS who developed MM were HHV-8 negative, in contrast to two patients with antibodies to HHV-8 who had not exhibited MM symptoms after 36 and 48 months. MacKenzie et al. [153] and Parravicini et al. [154] found only 2/78 and 1/20 MM patients to be seropositive to latent antigen, respectively. The presence of lytic antibodies in MM patients has also been difficult to find by other investigators. Utilizing recombinant ORF 65 antigen in ELISA and Western blot formats, MacKenzie et al. [153] and Parravicini et al. [154] found lytic antibodies in only 2/78 and 1/20 MM patients, respectively. Masood et al. [155] using a lytic IFA and a whole virus lysate ELISA found that only 2/28 MM sera were positive. Perhaps as the pathogenesis of HHV-8 becomes better understood this etiological question will be answered.

#### 5.E.c. Other diseases

Although there are many reports for other diseases and their possible associations with HHV-8, the data are sometimes circumstantial and weak, and many have not been confirmed by extensive investigation in large numbers of patients. Only a few selected diseases or conditions variably associated with HHV-8 are summarized below.

Bone marrow failure is a non-neoplastic disease possibly associated with HHV-8 observed after kidney and autologous peripheral blood stem cell transplants. HHV-8 produced a syndrome of fever, marrow aplasia and plasmacytosis; these occurred after primary infection and reactivation, respectively [104]. Neither patient presented with KS, but both had HHV-8 sequences detected by PCR after transplantation and at the presentation of symptoms.

HHV-8 infection has been associated with congestive heart failure in both KS and PEL patients [138]. Serological evidence has also indicated that Italian patients with cardiovascular disease have a higher prevalence of HHV-8 and HHV-8 DNA has been found in atheromatous plaques [156]. Other studies have suggested possible associations with HHV-8 and pemphigus vulgaris and pemphigus foliaceus [157] and germinotropic lymphoproliferative disorder [158], but not primary central nervous system lymphomas [159].

### 5.F. Treatment of HHV-8 infection

No single treatment has been found to be completely efficacious for HHV-8 infection. Anti-herpetic drugs such as foscarnet, ganciclovir, cidofovir, and acyclovir inhibit the viral DNA polymerase [107] which, therefore, only allows treatment for replicating viruses in the lytic phase of infection; latent viruses are unaffected. For example, although cidofovir was effective in vitro against BCBL-1 cells [160], intralesional injections were not helpful in reducing the KS tumor burden [161].

Chemotherapy and/radiotherapy are successful treatments for CKS but HHV-8 DNA has been shown to remain at the site of the healed lesion [162]. This might explain the observed reoccurrences of CKS. Treatment for AIDS-KS has centered on HAART. Studies have shown marked decreases in the incidence of AIDS-KS since the use of HAART [163]. However, this reduced risk has been only with triple therapy, and not double or single anti-HIV drug therapy [163]. Additionally, HAART seems to have the best effect on early stage AIDS-KS [164,165]; nonetheless, an 81% reduction in death due to AIDS-KS was observed though HAART [164].

Finally, because HHV-8 can be transferred from organ donor to recipient, the possibility exists that CTLs derived from the donor can be harnessed to provide immunotherapy for the recipient [100]. This has been shown to be an effective treatment for PTLD in the context of EBV reactivation after bone marrow donation [166].

## 6. HHV-8 Epidemiology

### 6.A. Serologic prevalence of HHV-8 geographically and in major risk groups

The serologic prevalence of HHV-8 infection has been explored in most continents worldwide and in different populations at different levels of risk of HHV-8 infection. It should be noted that the comparisons of prevalence are limited by whether antibodies to latent or lytic HHV-8 antigens were detected and the test formats used.

#### 6.A.a. North America

Studies from populations from the North American continent have revealed large differences in HHV-8 prevalence between specific populations. Blood donors (BD) have been found to exhibit different levels of infection ranging from no detected infection [167] to as high as 15% [168], with more intermediate levels (~5%) found in most studies [34,59,79]. Individuals infected with HIV infection or having AIDS had more elevated prevalences of 30%–48% [34,74,167], although one study found no evidence of HHV-8 infection in their small HIV cohort [167]. Homosexual men showed prevalences ranging from 20%–38% [74,169,170]. In contrast, the highest prevalences, between 88% and 100%, were found in those patients with KS [34,79,167]. Other miscellaneous populations, such as healthy individuals, the elderly, and those infected with EBV showed a range of 0%–8.6% [74,167,171]. IVDUs had relatively higher prevalences of 10% in both heterosexual men and women; the longer the patient's injected drug use, the higher was the risk of HHV-8 infection, which was not dependent upon sexual behavior or demographic differences [169]. Of note is the exceptionally high level of infection found in children in south Texas, 26% [172]. One report from Quebec, Canada, did not find evidence of HHV-8 infection in 150 renal transplant patients [173].

#### 6.A.b. The Caribbean and Central America

The prevalence of HHV-8 in BDs from Jamaica, Trinidad, and Cuba was 3.6%, 1.2%, and 1.2%, respectively [34,174,175]. Persons with HIV infection from Trinidad, Honduras, and Cuba possessed prevalences of infection at 0%, 24%, and 21%, respectively [34,175,176]. Compared to other studies in KS patients, a relatively low prevalence of HHV-8 infection was found in AIDS KS samples from Cuba (78%) [175]. A very low level of infection was found in attendees of a gynecology clinic in Jamaica (0.7%) [174], but an elevated prevalence was seen in healthy individuals in Honduras (11%) [176]. Commercial sex workers in Honduras showed 19% infection [176].

#### 6.A.c. South America

Evidence of HHV-8 infection has been discovered in South America in at least four countries. In indigenous populations, those without specific risk factors, prevalences of 53% were found among Brazilian Amerindians [177], 16% in northern Brazil [178], and 36% in Amerindians of Ecuador [179]; the prevalences in Ecuador ranged from 20%–100% depending upon the tribe tested [179]. The HHV-8 prevalence was much less in BDs in Brazil (2.8%), Chile (3.0%), and Argentina (4.0%); although in Argentina the prevalence in BDs ranged between 2.4% – 4.3% in three different locales [180]. In contrast, Sosa et al. [181] reported that in Argentinean HIV+ IVDUs, 17.4% showed HHV-8 seropositivity; where as, in HIV negative IVDUs the prevalence was lower at 11.1%. Still lower, HIV negative heterosexuals with no IVDU behavior had a prevalence of 5.7%, similar to that found by Perez et al. [180]. AIDS-KS patients in Brazil had a prevalence of 80% [182].

#### 6.A.d. Europe

In Europe, excluding Italy and its surrounding islands, the prevalence of HHV-8 in BDs was not above 6.5% in six countries: Hungary 0.83%–1.6%, Switzerland 5%, the United Kingdom 1.7%, France 2%, Spain 6.5%, and Germany 3% [59,83,183-186]. In healthy individuals in Switzerland, Greece, and Albania, evidence of HHV-8 infection was 13%, 12%, and 20% [59,184,187]. Persons infected with HIV ranged from a low of 16% in women in Germany to a high of 31% in homosexuals in the United Kingdom [59,184,186]. Homosexuals in Spain however, had an 87% prevalence [185]. IVDUs and persons with STDs in the United Kingdom, Spain, and France showed prevalences of 3.2%–8.4%, 12%–17%, and 13%, respectively [59,185,188]. Similar to North America, the HHV-8 prevalence in patients with classic or endemic KS was 75%, 94%, and 100% in Hungary, Greece, and France, respectively [59,83,183]. The HHV-8 prevalence in AIDS-KS patients in Switzerland (92%), the United Kingdom (81%), France (80%), and Germany (100%) were similar to the prevalence of HHV-8 in classic KS in Europe [59,83,184,186]. IVDUs in the United Kingdom and Spain had prevalences of 0.0%–3.2% and 12%, respectively [59,185].

#### 6.A.e. Italy/Sardinia/Malta/Sicily

Estimations of seroprevalence in Italian BDs were confounded by the variable geographic prevalences and the type of antibodies being detected. Whitby et al. [189] showed that the overall prevalence in 747 BDs in Italy was 14%. However, when these individuals were segregated by North/Central Italy and Southern Italy, the levels of HHV-8 infectivity dispersed to 7.3% and 24.6%, respectively. Even in Rome, centrally located in the country, the prevalence in BDs varied from 2% of people with latent antibodies to 28% with reactivity to lytic antigens [190]. Other reports found prevalences in BDs to be between 3.5% to 18.7% [167,191,192]. In the general population of Sardinia [61], Sicily [193], and for the elderly in Malta [194], antibodies to HHV-8 were found in 11%, 20%, and as high as 54%, respectively. In Italy, those infected with HIV showed a 14% prevalence for latent antibodies, but as high as 61% for lytic antibodies [190]; an intermediate rate (25%) in HIV+ persons was observed by Calabro et al. [192]. In Sicily, 34.6% of HIV+ patients had HHV-8 infection [193]. In regards to other STDs, infections with syphilis were accompanied by HHV-8 infections with 37%–76% showing coinfection, whereas those free from syphilis infection only showed 11%–46% prevalence [190]. No significant differences were seen in persons with or without HCV infections, 10%–50% and 16%–47%, respectively [190]. Perna et al. suggested that the relatively low prevalence of HHV-8 in drug addicts in Sicily (16.6%) was indicative of the poor transmission of HHV-8 parenteraly [193]. Calabro et al. [192] observed 61.5% prevalence in HIV+ homosexuals in Italy, but this rate might have been confounded by the coinfection of HIV because Perna et al. found a lower rate in homosexual men, 32.6% in Sicily [193]. Even healthy adults in Sicily had an elevated prevalence beyond that found in BDs with 36.2% observed with HHV-8 infection [193]. For this central region of the Mediterranean, the prevalence of HHV-8 in CKS normally exceeded that of AIDS-KS. CKS in Italy and Sardinia showed evidence of infection in 95%–100% of patients. However, AIDS-KS were reported to have a much wider range of reactivity in HHV-8 tests: 71%–79% [167], 57.1%–100% [191], 67%–83% [190], and 100% [192] in Italy, and 100% in Sicily [193].

#### 6.A.f. Middle East

Healthy individuals in Israel were found to have a HHV-8 seroprevalence of 4.8% [195], whereas individuals with HBV infection seemed to be at an increased risk of infection (22% prevalence) [81]. Family members from these hepatitis patients also had increased prevalence of HHV-8 at 9.9% [81]. When Ethiopian immigrants to Israel were tested for antibodies against HHV-8, this unique cohort possessed an elevated presence of antibodies against HHV-8 [124]. Fifty seven percent of Ethiopians with HIV infection showed HHV-8 infection, whereas those without HIV had a lower prevalence of 39.1% (*P *= 0.03). Interestingly, despite the high prevalence of HHV-8 in the HIV+ individuals, in those with AIDS, the occurrence of KS was almost nonexistent (0.85%) compared to non-Ethiopian immigrants with AIDS (12.5%) [124]. Reports on HHV-8 prevalence from Egypt are scarce. Andreoni et al. showed data that in teenagers and young adults, 29% possessed lytic antibodies against HHV-8, but only 5% had latent antibodies [191].

#### 6.A.g. Asia – Southeast and Asia proper

Blood donors and healthy individuals in five Asian countries have shown a 3-fold range in HHV-8 prevalence. In healthy Indian individuals [34], only 3.7% had antibodies, with Thailand, Malaysia, and Sri Lanka exhibiting prevalences no higher than 4.4% [34]. In Taiwan, lytic antibodies were found in 11.7% and 13% of the blood donors tested [196,197]. However, a much higher presence of prior infection was found in the general population of the Uygur people in northwestern China, 47% [198]. The prevalence of infection in HIV positive individuals in Asia varied widely, as well. Prevalences of HHV-8 infection of 0.6% to 11.2%, 2.4%, and 40% where found in Thailand, India, and Taiwan, respectively [34,197,199]. Classical KS still had the highest rate of infection, with 83% of patients in Taiwan [197] and 100% in China [198] showing positivity for HHV-8 antibodies.

#### 6.A.h. The Pacific region

There have been few studies on the seroprevalence of HVV-8 antibodies in the Pacific region. Despite this, the viral infection has been found in both Japan and New Guinea [200,201]. Fujii et al. [200] found a very low prevalence of HHV-8 infection in Japan in BDs where only 0.2% showed reactivity to latent antigen. Comparatively, persons with HIV infection had an elevated prevalence of between 9.8% and 11.6%. In New Guinea, Rezza et al. found a much higher prevalence in the indigenous general population with approximately 25% of the 150 people tested showing prior infection [201].

#### 6.A.i. Sub-Saharan Africa

In sub-Saharan Africa, the seroprevalence of HHV-8 was above 36% in every population reported. In the southern part of the continent, healthy individuals showed a HHV-8 prevalence of 37.5% in Zambia [34], and 54.7%–90% in Botswana, depending upon the test used [179,194]. In Zambia, the HHV-8 prevalence was comparable for HIV+ persons (44%) [34] and 51.1% in HIV+ pregnant women [202]. Cancer patients, in general, in South Africa also had a high prevalence of 36.3% [203]. In comparison, patients with AIDS-KS exhibited a prevalence of 83% in South Africa [203] and 92.3% in Zambia [202].

Central African nations also had HHV-8 prevalences in keeping with those observed in the south. In the Congo, a high prevalence in healthy individuals, 69%–79%, showed prior HHV-8 infection [194]. Somewhat lower percentages were found in healthy individuals in Ghana (41.9%) [34], in Uganda (38.7%) [34], 51%–62% [167]), and 55.5% in Cameroonian pregnant women [83]. Similar HHV-8 prevalences were found for HIV+ persons in Uganda with between 45.7% and 71% HHV-8 prevalence reported depending upon the study and the test used [34,59,167]. The prevalence of HHV-8 infection in AIDS KS patients was relatively higher but did not reach 100%; in Uganda, Gao et al. reported 78% and 89% [167] and Simpson et al. found 82% prevalence [59].

In conclusion, prevalence rates varied depending on the geographic origin of the sera tested and the specific tests used to determine these prevalences; in particular, whether antibodies against latent or lytic antigens were detected could make a difference in the results. Additionally, it is unclear whether these differences were truly due to varying prevalence rates, or perhaps to a lack of sensitivity and specificity of the serologic assays, as has been shown for HIV [204] and HCV [205]. Because most reports indicated high rates of HHV-8 infection in persons with KS, regardless of their origin, it is probable that the assays possess reasonable ability to detect true infection.

#### 6.A.j. Risks of age related HHV-8 infection

Regamey et al. reported that there was a trend of increasing HHV-8 antibody prevalence to Orf 65 antigen with increasing age in HIV negative individuals in Switzerland [184]. Below 30 years of age, the prevalence increased from 15% to 23% and then to 50% in the next three decades. A similar effect was observed in BDs in Hungary [183]. As age increased from 19 until 25 years of age and then for every decade afterwards, the distribution of seropositivity to LANA increased moderately, but significantly (*P *= 0.048). A similar association was observed with Orf 65 peptide reactivity but the numbers of subjects were too small to calculate statistical significance [183]. In Taiwan, increased progression of antibody response against HHV-8 lytic antigens was observed, starting with a low of 3% in children under five years of age and peaking between age 31 and 40 (19.2%) [196]. Many more examples of this have been reported in Africa [83,128,203], Sardinia [61], and Italy [192]. Perna et al. [193] and others [172,183,185,192] have shown that there most likely exists non-sexual routes of HHV-8 transmission because children worldwide have been infected by HHV-8.

### 6.B. Molecular prevalence of HHV-8 genotypes and variants

From DNA sequence analysis of distinct loci derived from 60 HHV-8 isolates, the clustering of four major HHV-8 viral subtypes was discovered [206]. These subtypes, A, B, C, and D are based upon DNA sequence derived from the K1 gene, a glycoprotein with transforming properties [207,208], and they exhibit 30% amino acid (aa) variability. These aa substitutions result from an 85% nucleotide substitution rate in this highly variable gene. The four subtypes were further divided into another 13 clades by Hayward [206]. The A1, A4, and C3 variants were predominant in the US AIDS KS samples, but the B variant was predominant in samples from Africa. C variants were observed from samples from Saudi Arabia and Scandinavia. The D subtype was uncommon and was found only in classic KS patients in the Pacific region. Another gene, K15, showed two different alleles (P and M), but these allelic types were not associated with the K1 subtypes [206]. These different genotypes have been investigated to explain the possible pathogenic and epidemiologic variation seen with HHV-8 infection [125]. Studies that are more recent have expanded upon previous work and have shown that the K1 locus can be divided into six subtypes with 24 clades showing strong linkage to the geographic origin of the particular isolate. Data have shown that subtypes A and C are prevalent in Europe, the U.S.A., and northern Asia. Subtypes B and A5 predominate in Africa and the D variant is found in the Pacific. Subtype E has been discovered in Brazilian Amerindians and a unique subtype Z was found in Zambia [125]. In a recent study, Whitby et al. characterized the K1 hypervariability from general populations in South America and Africa: i.e., those without any obvious symptoms of HHV-8 infection [179]. Amerindians from Ecuador carried the E subtype, in keeping with previous studies from South America. In Botswana, subtypes B and A5 were exhibited by subjects from the Bantu and San tribes, similar to the subtypes found there from KS patients. These results show that the same HHV-8 viral strains from similar geographic regions can be found in both diseased and non-diseased individuals, suggesting that there is no association between certain genotypes and disease.

## 7. HHV-8 Gene Products of Diagnostic Importance

### 7.A. Orf 73 (LANA1) latency protein

Immunofluorescent observations that PEL cells exhibited a distinct nuclear immunofluorescence after challenge with antisera from KS patients, led to the identification of Orf 73 as the gene responsible for the latency associated nuclear antigen-1 (LANA1) [209-211]. Early gene alignments had suggested that Orf 73 was an immediate early gene with 51% similarity to the Orf 73 of HVS [19]. Studies have since shown that LANA1 is a 222–234 kDa protein that is expressed in the majority of nuclei in KS spindle cells [211,212]; however, the LANA1 protein expression is variable [211] and can depend upon the clinical stage of the KS tumor [213]. The immunodominant epitope has been mapped to the C-terminal domain of the protein [210]. The gene is under latent control as evidenced by reduction in Orf 73 mRNA after chemical induction of the viral lytic phase [210]. The antigenicity of the recombinant LANA1 protein has been shown by Western blot; over 70% of HHV-8 IFA seropositive sera were LANA1 positive in the Western blot [210,211].

### 7.B. Orf 65 capsid protein

Orf 65 was identified by Russo et al. [19] as a lytic capsid protein with less than 60% similarity to similar capsid proteins from HVS and EBV, but is not cross-reactive with HVS and EBV capsid proteins [59,214-216]. Orf 65 has been shown to be the smallest component of the HHV-8 capsid with a predicted basic isoelectric point of 9.6, similar to other herpesviruses [217]. Because of its embossed structural position on the capsid, Orf 65 might be involved in interactions with the viral tegument and cellular proteins upon infection [218]. First cloned in bacteria by Simpson and colleagues [59] and subsequently by others [215], Orf 65 is a highly antigenic 18–22 kDa protein against which more than 81% of KS patients are seroreactive [59,215]. The dominant eight amino acid epitope has been mapped to the C-terminus, and allowed development of a peptide assay with reactivity in 90% of the KS samples tested [216].

### 7.C. K8.1 glycoprotein

Originally identified as a single gene locus [19], research has since shown that K8.1 is derived from spliced transcripts [219] for which the transmembrane sequence is appended [220]. This glycoprotein is unique to HHV-8 and is a TPA-inducible lytic protein [221]. On Western blots from induced PEL cells, it measures between 35–40 kDa with the characteristic smear of a glycoprotein [221]. Immunoelectron microscopy suggests that the virion acquires the K8.1 glycoprotein at the cell plasma membrane while budding from the host cell [222]. Two transcripts are produced, K8.1A and K8.1B, of which K8.1B is the shorter by dint of an internal deletion of 61 amino acids. K8.1A, casually referred to as K8.1, is very antigenic, with 97% of HIV+, KS+ patients having antibodies directed against it on Western blot; in HIV+, KS-, persons 61% showed reactivity [219].

### 7.D. Other antigenic proteins

Orf 25 and Orf 26 code for other major and minor HHV-8 capsid proteins, respectively, and were investigated for their diagnostic utility [223]. Orf 25 possesses 68% identity to the EBV BCLF1 major capsid protein and exhibited considerable cross-reactivity to EBV+ sera and was not used further in their studies. However, Orf 26 has only 49% identity to its EBV gene homologue and showed no cross-reactivity [223]. Only one third of KS patients were reactive to Orf 26, although some exhibited an increase in IgM and IgG reactivity 15 months prior to KS disease.

The Orf 59 protein is another HHV-8 protein that has shown modest diagnostic importance in a few investigations. This gene has about 50% similarity with its HVS and EBV homologues and is presumed to be a DNA replication protein in those viral systems [19]. Orf 59 is a 50 kDa protein with characteristic early-late lytic expression patterns seen for other viral proteins necessary for viral DNA replication [224]. The protein has been localized to the nuclear membrane via IFA and is observed in approximately 30% of induced PEL cells, but in less than 8% of uninduced cells [224]. The Orf 59 gene product, processivity factor-8, has been shown to be present in AIDS-KS tumors (50%) although perhaps not in as many spindle cells as Orf 73 [225]. Approximately 30% of AIDS-KS patients had antibodies against this antigen [226]. Orf 59 might be helpful in identifying aggressive KS disease [225,226].

## 8. HHV-8 Diagnostics

### 8.A. HHV-8 serological diagnostics

Presently, the diagnosis of KS requires clinical and histologic evaluation; however, the increasing documentation of its association with HHV-8 has raised the important possibility of being able to predict disease occurrence by demonstrating HHV-8 infection [55]. Additionally, there is a need to develop sensitive and specific serological assays to detect antibodies to HHV-8 for possible blood bank screening, assisting in clinical diagnosis, and in research to facilitate the understanding of the scope of this virus's association with rare, but nonetheless life threatening malignancies. HHV-8 infection can be identified by polymerase chain reaction in tissues and in cells; however, amplification methods are expensive, time consuming, and have been shown to be lacking in sensitivity for easily accessible diagnostic specimens such as plasma and PBMCs [177]. Alternatively, the testing for specific antibodies to HHV-8 offers a simple, inexpensive, and effective means to document infection and a help to define the relationship between infection and disease progression and yield insight into pathogenic mechanisms.

Currently, four methods have been used to demonstrate antibodies to HHV-8: enzyme-linked immunosorbant assay (ELISA), immunofluorescent assay (IFA), Western blot, and immunohistochemistry (IHC). Detection of infection and determination of seroprevalence can be dependent upon which test is selected [227]. ELISA methods vary according to the HHV-8 antigens used and whether they are recombinant antigens, viral lysates, or synthetic peptides. IFA methods incorporate virally-infected cell lines, either latently infected with expression of LANA1, or cells that express lytic antigens following chemical induction (i.e., those representing viral replication). The Western blot technique utilizes electrophoretically separated virally infected cell lysates or whole viral lysates, with transfer to nitrocellulose and then subsequent detection of reactive antigens; it has the advantage of identifying the presence of antibodies to specific antigens. IHC on fixed cells and tissue allows to determination of which cells harbor the virus in vivo and semi-quantitative analysis of infected sell type to help learn more about pathogenesis. IHC is also useful to confirm or rule out the clinical diagnosis of KS. These tests are explored in the following sections.

#### 8.A.a. HHV-8 antigen sources

PEL cell lines have been important sources of antigen mainly for use in IFAs, but also in the form of cell lysates for Western blotting and tools for investigations into HHV-8 pathogenesis [59,210-212,215,224,226]. Over 12 PEL cell lines have been established and they each contain 50–150 episomal copies of HHV-8 per cell [8,132-136]. About half are coinfected with EBV (e.g., BC-1, BC-2, BCBL-2), but others have only latent HHV-8 infection (e.g., BCBL-1, BC-3, KS-1) [135]. Induction of viral replication can be initiated by sodium butyrate (butyrate [228], 12-O-tetradecanoylphorbol-13-acetate (TPA), a phorbal ester [229], or less commonly hydrocortisone [130]. Cell cultures derived from KS spindle cells are not good material for HHV-8 diagnostics because they lose the virus after 2–6 passages [230].

Other sources of antigen have been whole virus lysate, which has been used successfully in the ELISA format [231]. After induction of a PEL cell line, the whole virus is usually purified over a sucrose gradient. The drawback of this method is that it preferentially selects for lytic antigens and does not allow detection of latent antibodies such as LANA1 [112]. In contrast, individual HHV-8 proteins have been incorporated into tests by either expressing them as recombinant proteins or as synthesized peptides. Recombinant proteins such as Orf 65, K8.1, Orf 25, and Orf 26 have been expressed in easy to grow bacterial systems [59,221,223]. Antigenic proteins have also been expressed in more difficult to grow baculovirus systems (insect cells) [232,233], but they have the added benefit of protein glycosylation which bacterial cells can not perform. It has also been reported that LANA1 (Orf73), because of its large size (>200 kDa) is expressed better in insect cells (personal communication, Dr. D. Whitby, NCI-Frederick). Synthesized peptides of immunodominant portions of antigenic proteins (e.g., K8.1, Orf 65) have been developed as a strategy to streamline the production process and to reduce non-specific reactions [183,234].

#### 8.A.b. ELISAs for the detection of HHV-8 infection

ELISA tests are easier to manipulate and technically are the test of choice for large-scale seroprevalence studies. ELISAs based on recombinant antigens of HHV-8 have shown that a specific humoral response is produced against capsid proteins of HHV-8, allowing identification of HHV-8 infection [59,92,223]. Recombinant proteins derived from a truncated Orf 65 minor capsid gene have been used with a relatively high degree of success to differentiate populations of KS patients from BDs [214]. Similarly, recombinant proteins derived from the Orf 25 and Orf 26 genes (major and minor capsid proteins) have been used in ELISA assays to detect IgG and IgM antibodies, but with a lesser degree of success [223]. Seroconversion against capsid proteins has been shown to occur in less than one year after infection using an Orf 65 ELISA [92].

An ELISA based on viral lysate antigens of HHV-8 has also produced encouraging results [231]. Although this assay demonstrated a good sensitivity for detecting infection in patients with classical KS (CKS) and AIDS-KS (80%–90%), normal healthy blood donors had 2–11% prevalence. This ELISA also possessed the ability to differentiate populations based on antibody titer; the mean titer in blood donors was 1:30, while titers ranged from 1:6000 to 1:15000 in AIDS-KS and CKS patients.

Encouraging results have come from a recombinant ELISA based upon the K8.1 gene product [235] and has been considered one of the more sensitive tests with acceptable specificity. Immunodominant peptides from the Orf 65 and K8.1 antigens were incorporated in an ELISA format and used successfully to measure the risk factors in women [76] and to identify HHV-8 infection in allogeneic stem cell transplant patients who are at risk of KS because of their immunocompromised status [236].

Initially, the primary method of detection of latent antibodies was using the LANA IFA, however, subsequent cloning of Orf 73 (LANA) and its application in the ELISA format has begun to replace the LANA IFA. The Orf 73 ELISA has been found to possess the same high specificity, but with a 10% increase in sensitivity [107]. The Orf 73 ELISA has found utility in gauging the progression to KS in HIV+, HHV-8 infected persons [43]. In that study, increasing titers to Orf 73 over time were associated with HIV+ patients acquiring KS.

#### 8.A.c. IFA for the detection of HHV-8 infection

IFAs are a common method to identify antibodies to HHV-8. To detect latent antibodies, an HHV-8 infected PEL cell line (e.g., BCP-1, BCBL-1, BC-3, KS-1) is used to measure antibodies to the primary latent antigen, LANA1 or ORF 73 [107]. This latent antigen corresponds to a ~234 kDa nuclear antigen, which has been shown to be recognized by sera from KS patients [211], and is characterized by its speckled nuclear fluorescent signature in 95% of PEL cells [107]. With this assay, seroprevalences have ranged from 2%–27% in several studies of blood donors where KS is endemic, but lower (0%–15%) for those geographic regions where KS is mainly associated with AIDS and transplant patients [227]. However, the LANA1 assay has been shown to be relatively insensitive and therefore might not be the best choice of assay to screen low titered populations [235].

Lytic antigens can be expressed by these cells following induction with a TPA or with butyrate [237] and have produced encouraging results. The number of induced cells is dependent upon the cell line used, the time of induction, and the chemical used to induce the cells [107,238]. Studies using induced PEL cell lines point to much higher frequencies of infection than have been suggested by serology based on latent proteins in populations not at risk for sexually transmitted diseases [16,239]. However, other studies using lytic IFAs have also indicated that there are higher levels of HHV-8 infection in otherwise healthy individuals [227] and infection would be spread by non-sexual routes in these cases. As with the ELISA, the IFA has been used to determine antibody titers, with sera from HIV-positive persons with KS demonstrating higher titers to lytic and latent antigens as compared to individuals without KS [214,231]. This test method is relatively more sensitive to serum dilutions that are not extensive enough; the correct serum dilution is important to correctly differentiate true positive reactions from those that are non-specific [112,214].

#### 8.A.d. The diagnostic utilities of the Western blot

Western blots using purified viral lysates of HHV-8 have been used to identify immunodominant proteins using sera from pre- and post-KS patients [114,221,240]. This method has shown utility in the diagnosis and prognosis of KS, but it is more cumbersome and expensive than other serologic assays. A 35–37 kDa glycoprotein has been a protein most frequently and intensively detected, and corresponded to the K8.1 Orf of HHV-8 [220].

In a review of articles that used Western blot in their investigations of HHV-8, only four dealt with antigen identification or expression. These reports could influence the development of a confirmatory Western blot as they showed: 1) There are different antigen profiles in diseases associated with HHV-8 [140]. 2) HHV-8 possesses the glycoprotein, gB, found in other herpesviruses and might be a candidate antigen [241]. 3) That different risk groups and different stages of disease could exhibit different antibody profiles [242]. 4) Patients undergoing antiviral therapy might not produce certain antibodies due to a decreased expression of HHV-8 antigens [238]. These findings suggest that it might be necessary to identify specific antigens for use at specific times of infection and even for different disease states.

Nine reports involved the use of Western blots for screening purposes [61,94,210,219,221,226,243-245]. Recombinant Orf 65 was used most often followed by K8.1, Orf 59 and Orf 73, and finally vIL-6 and Orf 47; however, there was no utility in using vIL-6 or Orf 47. In these reports, KS sera were detected by K8.1 with the greatest sensitivity, followed by Orf 65 and then Orf 73. In these studies, there were not sufficient HIV+ sera examined to draw conclusions as to which antigen was best in that specific population. Sera/plasma from healthy controls varied from a low of 0% for Orf 59 and Orf 73 to 6.5% for K8.1 and 8.3% for Orf 65.

Eleven research reports utilized Western blots as tools to confirm the results of previously run serological assays [59,92,183,232,246-252]. Most of these authors used the same antigen found in the ELISA as the confirmatory antigen in the Western blot; however, two reports had the Western blot confirm IFA results. In seven instances, the authors used the Western blot to confirm a single screening assay and in four reports, they used the Western blot it to resolve a disagreement between two screening assays or in duplicate samples.

More recent reports have continued to use the Western blot as both a primary assay and as a confirmatory test [175,253]. The Western blot method has the benefit of allowing identification to one or more antigens. With accessibility to multiple recombinant proteins now possible, several researchers have developed recombinant Western blot utilizing more than one protein [197,254]. In those reports, they accepted reactivity to one of three antigens to be a marker of HHV-8 infection. In this manner, Wang et al. proposed a new antigen, Orf 57, for use in asymptomatic populations [197]. Clearly, despite the technical difficulties in producing Western blots, they are a useful, multitasking serological method in HHV-8 diagnostics.

#### 8.A.e. Comparisons and concordances between assays

Estimates of the prevalence of HHV-8 by different ELISAs have varied. This variance has been shown in reports of multicenter or multitest studies. Spira et al. [255] found a range of concordance between 69% and 94% using seven serologic tests with Kappa values as low as 0.387 (fair agreement) and as high as 0.909 (almost perfect). Rabkin et al. [256] also evaluated seven serologic tests and found a range of concordance between 50% and 94%, with Kappa statistics ranging from -0.08 to 0.86, indicating that the interassay correlation between the assays was less than favorable. The tests frequently disagreed on individual sera, particularly from blood donors. It was concluded that current antibody tests for HHV-8 have uncertain accuracy in asymptomatic HHV-8 infection and that additional tests to define the actual prevalence may be required. Poor correlation for positive results has been observed in other studies [257]. Second generation tests seem to provide better concordances, although the best results came from IFA tests rather than ELISAs in one multicenter study [258]. Even with more optimized assays, sensitivity and specificity can be insufficient for clinical use [235]. As with the detection of infection by many viruses (e.g., HIV), sequential use of screening and confirmatory tests for HHV-8 are likely to be required to address sensitivity and specificity issues; accordingly, an testing algorithm has been reported [235]. These findings supported the need for critical investigation of the parameters that could influence the performance of these tests.

Although there is some variability in prevalence among similar populations with the same test, most data show that there is agreement within a defined range. The lack of concordance in HHV-8 diagnostic assays occurs primarily because not all HHV-8 infected persons exhibit all antibodies against all HHV-8 antigens at the same time [259]. This phenomenon of single antibody reactivity is much more apparent in populations who are at low risk of infection, such as blood donors [259]. Because of this, specificities are more variable than sensitivities among different laboratories [259].

Although refinement of the diagnostics assays is still possible, the greatest chances of success are in developing algorithms that make use of multiple assays for screening and then confirmation or alternatively, the use of assays that incorporate multiple antigens which have been shown to be highly immunogenic, perhaps during different stages of infection [259]. It is possible to use a combination of latent and lytic antigen tests to determine a true positive as has been employed by several laboratories [112]; however, recent data indicate that the humoral response to HHV-8 does not always produce both latent and lytic responses at the same time [260]. In addition, antibodies directed against lytic antigens seem to be more prevalent than those for latent antigens.

#### 8.A.f. IHC for the detection of HHV-8 infection

IHC is a powerful serologic tool, but like Western blots can be tedious to perform. In the field of HHV-8 research and diagnostics, IHC has been used to locate HHV-8 proteins, assess involvement of HHV-8 in malignancies, detect specific HHV-8 gene expression, and to provide diagnosis of KS. The ability to identify which specific cell types or structures within a cell are expressing HHV-8 proteins can assist in the understanding of HHV-8 pathogenesis [261,262] and determine the possible etiology of malignancies [263-267]. Detection of specific HHV-8 gene expression, in particular LANA1 [140,211], has led to possible clinical applications for the diagnosis of KS in tissue samples [268,269]. This allows the exclusion of other neoplasms that can mimic KS [268-270]. The ability of monoclonal and polyclonal antibodies to localize specific HHV-8 antigens should continue to improve HHV-8 diagnosis and our understanding of HHV-8 pathogenesis.

### 8.B. HHV-8 molecular diagnostics

The diagnostic benefit of the polymerase chain reaction (PCR) for herpesviruses other than HHV-8 has been mixed. Studies have shown a lack of correlation with PCR and positive serological tests results for viral retinitis [271] and no herpesvirus sequences were discovered in the PBMCs of suffers of chronic fatigue syndrome (CFS) [272]. Other studies, however, have found PCR to be useful in diagnosing HHV-6 infection in exanthum subitum during convalescence where IgM is no longer detected [273]. In general, Pearson et al. recommended the use of PCR to better diagnose acute infection or reactivation in herpesvirus infections unless sentinel antigens could be identified [274]. For the detection of HHV-8, the PCR method with optimal performance should fulfill several conditions. The test should be specific for DNA sequences found only in the HHV-8 genome and not other herpesviruses. The K-genes might be good candidates for this, and indeed a real-time PCR test using the K6 region has been used [177]. Sensitivity is an absolute requirement because the virus is found at such low copy numbers due to its latent biology. Most reports have indicated sensitivities from 1–100 copies per reaction [275]. However, the herpes-specific biology makes sampling error a concern. Therefore, strategies are needed to detect the virus in latency, such as induction of the lytic cycle before DNA isolation. There have been a few reports where this has been attempted with success [48,58]. If nested PCR is to be used to gain the needed sensitivity, exceptional care must be taken to avoid false positives. However, nested PCR has the power to provide added specificity and confirmation by amplifying two separate amplicons in the nested PCR reaction. Alternatively, multiplexing in real-time PCR, with the proper optimization and design, could provide this needed level of surety. An easily obtainable diagnostic sample would complete the diagnostic strategy to maximize the effectiveness of PCR for the detection of HHV-8. Reports have shown that saliva contains the highest prevalence of virus in HIV negative persons [56] and in samples from HIV+ patients it is equivalent to PBMCs [51,56]; therefore, it should be considered the sample site of choice. Saliva collection devices are already commercially available (OraSure, Bethlehem, PA) and FDA approved for serologic testing and might be convertible for use for PCR. Finally, the ability to quantify HHV-8 viral loads using quantitative real-time PCR has been employed to measure HHV-8 viral burdens to investigate the association of viral load and progression to KS [276,277] and the pathogenesis and transmission of HHV-8 [86,278].

In a review of the literature, the use of molecular diagnostics, in particular PCR, for the detection of HHV-8 infection has been less than optimal. In most cases, serology is the preferred method to identify HHV-8 infection. Most articles have shown that at least one serological assay had better sensitivity than PCR on the same samples, even better than nested PCR. For example, in a study of AIDS-KS, IFA was able to detect HHV-8 antibodies in 50% (latent) to 100% (lytic) of the patients, whereas, nested PCR detected infection in only 33% [57]. The data from a minority of reports showed that PCR was a more favorable assay in isolated cases [279] or that serology and PCR were comparable [280]. In a composite set of 642 samples from numerous reports, 69% were concordant in their PCR and serology results. However, 179 samples (28%) were positive by serology, but PCR negative; only in 21 samples (3.3%) was there a PCR positive result without a corresponding positive serology.

The utility of PCR in detecting HHV-8 in KS patients is better but not perfect. PCR appears to be very useful when detecting HHV-8 directly in the KS lesions, with sensitivity approaching 100% [50,51,183,279]. However, PBMCs from KS patients were observed to have fewer instances (~50%) of detectable viral sequences [50,51,55]. Detection of HHV-8 DNA by PCR in the PBMCs of HHV-8 infected individuals is not a common event. Only 10–20% of seropositive persons have detectable HHV-8 DNA, but this percentage increases with evidence of KS disease and more severe disease [259]. Even in KS lesions, if the tissue sample is not processed correctly for PCR, there can be false negative results [259]. However, PCR has been found to be useful in detection of early infection or reactivation, especially at times of clinical sequelae of viral primary infection or reactivation [49].

Few reports have used plasma or sera as the analyte for PCR, especially juxtaposed to serological methods [49,56]. However, these investigators seem to indicate it does not perform any better than PBMCs. It is noteworthy to add that several authors observed HHV-8 viremia to be intermittent. In longitudinal samples, several investigators have found that despite enhanced detection schemes and serial samples over periods of time exceeding two years, detection of HHV-8 in PBMCs can be missed 30% of the time or more [54,57,58,281,282]. Even in saliva, which has been shown to carry a relatively higher viral burden, due to intermittent shedding up to 65% of the time, detection of the virus can be missed if only single samples are relied upon for diagnosis [51,56]. Finally, in serum/plasma, detection of can be intermittent with perhaps the best chance of detection at signs of clinical disease [49,54].

Reports on the use of in situ hybridization and reverse-transcriptase PCR (RT-PCR) have been used as mainly research tools to investigate associations of HHV-8 and specific diseases [29,267]. Most RT-PCR reports were concerned with detecting mRNA transcripts to determining infectivity [283] or as a diagnostic method in HHV-8 related disorders, such as PELs [284]. There have been few reports using nucleic acid sequence-based amplification (NASBA) assays to detect and quantitative HHV-8 viral loads in HHV-8 diseases [285,286], although the reports seem to confirm the findings from quantitative PCR studies that increased viral load in to be expected in more advanced KS, both in the lesions and in the PBMCs.

### 8.C. Commercial sources

Although HHV-8 has been associated with only a few diseases, commercial sources for both testing and kits are available. These include molecular and serologic testing from established laboratories and hospitals, although PCR seems to be the method most used (Table 2). IFA or ELISA serologic kits are also commercially available (Table 3), but no companies seem to be marketing molecular kits except for Celonex, which produces a microarray system for herpesviruses.

**Table 2:** Companies or institutions that provide molecular testing services or research kits for the detection HHV-8 infection.

Molecular testing & kits		
**Company**	**Test**	**Utility**

**Focus Diagnostics, Inc**. Herndon, VA, USA	PCR, qualitative	Method for identifying individuals among HIV+ persons who are at increased risk for developing KS. "The results are for research use only, and should not be used for diagnostic purposes."
**ViraCor Laboratories **Lee's Summit, MO, USA	Real-time PCR, quantitative (100 copies/ml to 1 × 10^10 ^copies/ml)	Clinical diagnostics: Determination of HHV-8 primary infection and for determining the risk of developing KS among organ transplant patients and patients taking immune suppressive drugs.
**ARUP Laboratories **Salt Lake City, UT, USA	Real-time PCR, qualitative (limit of detection: 1 in 100,000 cells)	Clinical diagnostics: To predict the development of KS, to aid differential diagnosis in other vascular neoplasms and inflammatory conditions that are histologically similar to KS, to diagnose PELs, and to monitor patients with immune compromise or dysregulation.
**LabPLUS **Auckland City Hospital, New Zealand.	PCR, qualitative	Clinical diagnostics: Diagnosis in KS, PEL, MCD
**Medical Diagnostic Laboratories, L.L.C. **Hamilton, NJ, USA	PCR, qualitative	Clinical diagnostics
**UT Southwestern Medical Center **Dallas, TX, USA	Real-time PCR, qualitative	Clinical diagnostics
**Celonex **Edmonton, Alberta, Canada	Single HHV-8 ViruChip™	Gene expression

**Table 3:** Companies or institutions that provide serologic testing services or research kits for the detection HHV-8 infection.

Serologic testing & kits		
**Company**	**Test**	**Utility**

**Fred Hutchinson Cancer Research Center **Seattle, WA, USA	ELISA	Clinical diagnostics
**Focus Diagnostics, Inc**. Herndon, VA, USA	IgG IFA	Method for identifying individuals among HIV+ persons who are at increased risk for developing KS. "The results are for research use only, and should not be used for diagnostic purposes."
**Quest Diagnostic **(Focus Technologies) Baltimore, MD, USA	IgG IFA	"This test should not be used for diagnosis without confirmation by other medically established means".
**Advanced Biotechnologies Inc **Columbia, MD, USA	1) IgG Antibody IFA Kit 2) IgG Antibody ELISA Kit (whole virus lysate)	For research use only.
**Biotrin International **The Rise, Mount Merrion Co. Dublin, Ireland	1)IgG IFA assay 2) DIAVIR HHV-8 peptide mix (Orf 65 & K8.1A) ELISA	For research use only. To aid in the diagnosis of primary infection or to identify reactivation or reinfection. To determine current or recent infection by testing of paired specimens of plasma or serum taken 7–14 days apart; a ≥ 4-fold rise in titer is indicative of recent infection.
**Panbio Inc. **Columbia, MD, USA	1) IgG IFA (Lytic) 2) IgG IFA (Latent) 3) DIAVIR HHV-8 peptide mix (Orf 65 & K8.1A) ELISA	For research use only

## 9. Current Diagnostic Issues

Current HHV-8 diagnostic tests are not commonly used in the clinical arena because their procedures are not standardized and the specific patient populations to which they would best be applied are not clearly identified. Investigators have not been able to unambiguously determine if low risk individuals, such as blood donors, who happen to test positive using the current array of assays, are truly infected. Therefore, there is an urgent need for a gold standard, FDA-approved diagnostic test for HHV-8. The difficulty in detecting HHV-8 in patients makes development of a gold standard seroassay difficult at best and the determination of specificity almost impossible. The current, incomplete understanding of how HHV-8 is transmitted, and the risk factors associated with its transmission add to the burden of correlating diagnostic test results to true infection. For example, a patient admitted to an emergency room complaining of myalgia, fever, and headaches could be presenting with symptoms from any number of infectious or non-infectious illnesses. However, if the clinical history indicates a recent walk in the woods with a tick bite, then the diagnostic picture narrows to include the possibility of ehrlichiosis or borreliosis. The translation of research knowledge into the clinical arena will require careful development, evaluation, optimization, and refinement to develop a new standard of care that blends advances in both diagnostic and clinical sciences [287].

There are other deficiencies in HHV-8 diagnostic testing methods. First, there is no effective HHV-8 confirmatory assay similar to the Western blot used with HIV. Because of the large variability of results between current tests and between tested populations, it is difficult to find agreement between two tests, except perhaps, in KS patients. The inconsistent assay results also impede development of effective diagnostic algorithms. Second, the availability of an antigen capture assay (currently unavailable) would benefit HHV-8 diagnostics in several ways. For example, knowledge of the time course and concentrations of virus circulating in patients (temporal antigenemia) could help elucidate the natural history of HHV-8 infection, which in turn could be utilized to detect early HHV-8 infection, to confirm infection, and to monitor therapy. Suitable antigens with high copy number such as capsid proteins would be required. High affinity and high avidity antibodies would need to be identified or developed, and preferential access to the respective recombinant antigen would be required for test development and for use as test controls. Fortunately, commercial and research sources of antibodies exist against both latent and lytic antigens, such as LANA1, K8.1, Orf 65, and Orf 59, which will accelerate development of antigen assays.

There is a deficiency of HHV-8 antigenic proteins for use in diagnostic tests. Current HHV-8 ELISAs target IgG antibodies to one of three viral antigens: K8.1 [235], Orf 65 [59], or Orf 73 [235]. To date, no other useful HHV-8 proteins have been discovered that provide acceptable sensitivity and specificity in all populations tested, despite a viral genome that can express over 47,000 amino acids. Further research into identifying antigenic proteins is needed.

The use of Western blot as a screening tool for HHV-8 is impractical, and Western blot confirmatory tests suffer from nonspecific reactions when whole cell lysates are used. Currently, the choice of HHV-8 antigens is limited for development of recombinant immunoblots making the formulation of confirmatory Western blots difficult.

Although many published reports have confirmed the utility of antibody isotype tests other than IgG for the detection of other herpes viral infections, there is a dearth of reports detecting anti-HHV-8 IgA and IgM antibody isotypes. For example, patients with chronic fatigue syndrome and multiple sclerosis were more apt to have IgM antibodies against HHV-6 [288,289]. IgA against EBV VCA is at a higher seroprevalence and geometric mean titer in patients with EBV-positive gastric carcinomas [290], and is predictive of nasopharyngeal carcinoma [291]. This is in contrast to HHV-8 where there are few reports of HHV-8 antibody isotype assays for IgA and IgM [92,167,223,249,257,292], and none where the investigator compared IgG, IgA, and IgM isotypes concurrently in the same laboratory with the same tests and serum samples. Theoretically, detection of IgA and IgM anti-HHV-8 might improve identification of HHV-8 infection and provide early diagnosis. IgA and IgM isotype detection could also be incorporated into improved diagnostic algorithms to better define the prevalence and disease associations of HHV-8 infection.

If HHV-8 is similar to other herpesviruses, there may be difficulty in identifying specific antigens to which the majority of infected individuals have mounted an antibody response. For example, among the many other viral structural proteins of HCMV, only one, pp150, is recognized by most infected individuals [293] and a p101 protein was found to be most antigenic for HHV-6 [294]. Finding immunodominant antigens may take extended study and application of novel techniques. In addition, determining the sequence of specific antigenic gene products from viral isolates from diverse geographic regions is necessary to ensure that antigens used as a lure for HHV-8 specific antibodies are universally detected [216,295].

In regards to molecular testing, only a few reports have evaluated the utility and efficacy of performing PCR on activated PBMCs isolated from persons potentially infected with HHV-8. Cell culture activation of a blood donor's PBMCs using IL-2, TPA, and hIL-6 increased detection from 1/7 to 5/7 serial samples [58]. Another report showed that the presence of inflammatory cytokines, specifically Inf-γ, increased the HHV-8 viral load to detectable limits in cultured PBMCs derived from both AIDS-KS and non-KS AIDS seropositive patients [48]. Studies to confirm this seemingly useful approach and to define the optimal viral amplification procedures are needed.

The reverse transcriptase PCR (RT-PCR) assay is a popular molecular diagnostic test for retroviruses or RNA viruses, such as HCV or HGV. RT-PCR is usually not necessary for DNA viruses, because the viral genomic DNA itself can be detected without the intermediate step of reverse transcriptase to create cDNA. However, since the unique latent biology of HHV-8 renders DNA PCR of HHV-8 relatively insensitive, RT-PCR should be studied more thoroughly as an alternative diagnostic test for the detection of HHV-8 infection. The rationale is that detection of mRNA provides a built in preamplification step for detection of the viral nucleic acid, because mRNA is at a higher copy number than the corresponding genomic DNA. This method could also allow the detection of both latent and/or lytic transcripts increasing the chances of success. To our knowledge, there are no reports in the literature that RT-PCR has been evaluated seriously as a diagnostic or screening assay for HHV-8 infection.

As an adjunct to the necessity of improved HHV-8 diagnostics, the effective use of HHV-8 viral therapy will depend on the development of sensitive and specific HHV-8 diagnostic tests to gauge the therapy's effectiveness. Accumulating research either has implicated HHV-8 as the etiologic agent of diseases such as KS or has associated the virus indirectly with disease development. The efficacy of clinical therapeutic drug interventions for HHV-8 infection has not been studied thoroughly in clinical settings, rather, mainly through in vitro experiments. Prospective anti-HHV-8 therapeutic trials of anti-herpetic drugs are needed in large and diverse cohorts of AIDS patients presenting with KS. Organ transplant patients, in order to prevent organ rejection, also require intense study to determine the proper anti-HHV-8 intervention in the absence of HAART and in the presence of immunosuppressive therapy. It will be more difficult to study the therapy of patients with PEL and MCD because of the low prevalence of these diseases.

Modern medicine will be able to manage this novel human herpesvirus only through continued research into the dynamics of HHV-8 infection in vivo, and the identification of important and unique antigens and their subsequent development into diagnostics tests. Such advances in turn will result in better understanding of the pathogenesis and associated diseases of HHV-8 and catalyze antiviral therapy and strategies for prevention.

## 10. Conclusion

Although the prevalence of HHV-8 is not as ubiquitous as other human herpesviruses, there is strong evidence that it is required and quite possibly is the primary etiological agent for the formation of several life threatening neoplasms, including KS. Therefore, the development and optimization of improved diagnostic assays is critical for the identification, diagnosis, and monitoring of HHV-8 infection. Our work at the University of Maryland Baltimore has addressed important issues in the field of HHV-8 investigation; namely, the lack of a gold standard serologic assay to detect the virus or antibodies to the virus, a lack of optimization of current serologic assays, few reliable diagnostic HHV-8 antigens available for serologic tests, the epidemiology of HHV-8, and an incomplete understanding of the host humoral response to HHV-8 infection.
